# Targeting TKT-associated immunometabolic remodeling attenuates experimental lupus nephritis and NET-related inflammation

**DOI:** 10.3389/fcell.2026.1835407

**Published:** 2026-07-01

**Authors:** Litong Zhu, Taoyan Lin, Lai Yee Cheong, An Ying Christy Chui, Tak Mao Chan, Desmond Y. H. Yap

**Affiliations:** 1 Division of Nephrology, Nanfang Hospital, Southern Medical University, Guangzhou, China; 2 National Clinical Research Center for Kidney Disease, Nanfang Hospital, Southern Medical University, Guangzhou, China; 3 State Key Laboratory of Multi-Organ Injury Prevention and Treatment, Nanfang Hospital, Southern Medical University, Guangzhou, China; 4 Guangdong Provincial Institute of Nephrology, Nanfang Hospital, Southern Medical University, Guangzhou, China; 5 Guangdong Provincial Key Laboratory of Renal Failure Research, Nanfang Hospital, Southern Medical University, Guangzhou, China; 6 Clinical Pharmacy Center, School of Basic Medical Sciences, Nanfang Hospital, Cancer Research Institute, Southern Medical University, Guangzhou, China; 7 Division of Nephrology, Department of Medicine, Queen Mary Hospital, The University of Hong Kong, Hong Kong, Hong Kong SAR, China

**Keywords:** immunometabolism, lupus nephritis, neutrophil extracellular traps (NET), systemic lupus erythematosus, transketolase (TKT)

## Abstract

Lactic acid metabolism and neutrophil extracellular traps (NETs) are critical immune regulators, yet their specific crosstalk in systemic lupus erythematosus (SLE) and lupus nephritis (LN) pathogenesis remains poorly understood. Here, by integrating bulk and single-cell RNA sequencing (scRNA-seq) analyses, we identified *TKT* and *ITGAM* as pivotal upregulated genes in SLE, demonstrating robust diagnostic value (AUC >0.7) supported by reliable nomogram models. Additional analysis of a human LN renal biopsy dataset (GSE32591) showed increased ITGAM and TKT in LN glomerular samples, supporting their renal relevance. Regulatory network analyses suggested potential molecular interactions involving these genes with specific microRNAs (e.g., hsa-miR-142-5p-ITGAM and hsa-miR-1-3p-TKT), while scRNA-seq analysis suggested cell-type-associated expression patterns, with donor-level analysis showing higher TKT expression in monocytes from SLE patients. To translate these computational insights into biological relevance, we conducted rigorous *in vivo* validations. In an apoptotic cell-induced LN mouse model, we confirmed renal injury, increased ITGAM and TKT protein expression, and enrichment of TKT in CD14^+^ monocyte-lineage cells. Crucially, targeted pharmacological inhibition of TKT using oxythiamine significantly mitigated disease progression in LN mice. Oxythiamine treatment not only restored renal function and attenuated tissue fibrosis but also reprogrammed aberrant lipid metabolism and cellular proliferation. Oxythiamine treatment was further associated with reduced ITGAM expression and lower renal Cit-H3 levels, consistent with an attenuation of NET-associated inflammatory activity. Together, our integrated multi-omics and *in vivo* data indicate that TKT is closely linked to immunometabolic remodeling in experimental lupus nephritis, accompanied by alterations in lactate accumulation, ITGAM expression, and NET-associated inflammatory activity. These findings support TKT-associated metabolic remodeling as a potential therapeutic avenue that warrants further mechanistic investigation in LN.

## Introduction

Systemic lupus erythematosus (SLE) is a clinically heterogenous autoimmune disease that affects multiple organ systems. The pathogenesis of SLE involves genetic predisposition, environmental triggers, or hormonal dysregulation (Morand et al., 2023; Tsokos, 2020). Although immunosuppressive therapies have improved over time, the clinical outcomes often remain suboptimal and the treatments are still associated with considerable short- and long-term side effects. In addition, management of SLE poses chronic and significant economic burden to the healthcare systems (Siegel and Sammaritano, 2024; Yao et al., 2023; Bindra et al., 2023; Masurkar et al., 2024), resulting from expensive new therapies and the costs for managing treatment-related complications. Therefore, it is important to develop new biomarkers for better disease monitoring and more effective immunomodulatory medications with improved toxicity profiles. Enhanced understanding on the immunological mechanisms of SLE is prerequisite to the discovery of novel biomarkers and therapeutic targets.

Recent studies had reported the roles of lactic acid metabolism and neutrophil extracellular traps (NETs) in the pathogenesis of SLE (Sun et al., 2024; Psarras and Clarke, 2023; Shi et al., 2019). The metabolic pathway of lactic acid is a key player in energy and immune homeostasis in cells. Previous research suggested that cytosolic mitochondrial DNA (mtDNA) in SLE can induce glycolytic reprogramming in macrophages, subsequently enhancing aerobic glycolysis and lactate production. The accumulation of lactate promotes the lactylation of cyclic GMP-AMP synthase (cGAS), thereby suppressing its ubiquitination and degradation, and ultimately amplify the cGAS/STING signaling pathway to stimulate type I IFN synthesis. These observations have insinuated the role of lactate and lactylation in SLE development and progression (Zhang et al., 2024). Aberrant lactic acid metabolism could affect the immune cell activation and inflammatory pathway through intracellular pH modulation (Lin et al., 2023), and its dysregulation has been implicated in development and progression of lupus nephritis (LN) (Li et al., 2022; Hortová-Kohoutková et al., 2022). NETs are DNA clusters released from activated neutrophils, which are involved in both pathogen defense and autoimmunity (Wang et al., 2023). Abnormal NET formation in SLE can lead to tissue injury and persistent inflammation (Liu et al., 2023; Dömer et al., 2021). Recent evidence has also suggested a link between lactic acid metabolism and NETs, where metabolism of lactic acid may dynamically promote NETs release via their effects on neutrophil activation and the downstream signal transduction pathways (Sun et al., 2024; Angeletti et al., 2021).

Given this background, the present study aimed to elucidate the intricate interplay between lactic acid metabolism, NET formation, and immune infiltration in the pathogenesis of lupus nephritis (LN). To achieve this, we employed a highly integrated research paradigm, coupling comprehensive bioinformatic profiling with rigorous *in vivo* validation using an apoptotic cell-induced LN mouse model. Crucially, we extended our investigation beyond observational data by conducting targeted pharmacological interventions *in vivo*.

## Materials and methods

### Data source

The bulk transcriptomic datasets GSE50772 (61 SLE patients and 20 controls) (platform: GPL570) and GSE72326 (157 SLE patients and 20 controls) (platform: GPL10558), along with scRNA-seq dataset GSE135779 (7 SLE patients and 5 controls) (platform: GPL20301), were obtained from the Gene Expression Omnibus (GEO) database (https://www.ncbi.nlm.nih.gov/gds) (Kennedy et al., 2015; Li et al., 2023; Nehar-Belaid et al., 2020). In addition, the human LN renal biopsy transcriptomic dataset GSE32591 was included for renal tissue-level validation. This dataset contains microdissected glomerular transcriptomic profiles from 32 human LN renal biopsy samples and 14 control renal samples. The expression levels of ITGAM and TKT were compared between LN and control glomerular samples to evaluate whether the peripheral blood-derived candidate genes were also dysregulated in human LN kidney tissue. The GSE50772 and GSE72326 cohorts were used as training and validation sets respectively. Raw data for the GSE50772 training set and GSE72326 validation set were downloaded from the GEO database using the GEOquery package. Expression matrices were extracted, and missing values and invalid data points were removed. Probes were annotated to gene symbols according to the corresponding GPL platforms. Multiple-gene probes were split, and unannotated probes were discarded. For genes mapped to multiple probes, only the probe with the highest mean expression value was retained to generate the final gene-level expression matrix. Because the bulk datasets were microarray-based expression profiles rather than raw RNA-seq count matrices, conventional low-count gene filtering was not applied. Instead, probes with missing or invalid values, unannotated probes, and redundant probes were removed during preprocessing before generating the final gene-level expression matrix. Healthy control and systemic lupus erythematosus (SLE) samples were selected, and irrelevant samples were excluded to ensure consistent grouping. The gene expression matrices were finally normalized using the log2 (x+1) transformation. Because GSE50772 and GSE72326 were used as independent training and validation cohorts rather than merged into a single expression matrix, cross-dataset batch-effect correction was not performed. Adjustment for disease activity, treatment status, or sex imbalance was not performed because these clinical variables were not consistently available across the public GEO datasets.

Lactic acid metabolism-related genes (LAM-RGs) (Li et al., 2024) and neutrophil extracellular traps-related genes (NETs-RGs) (Gao et al., 2024) (comprising 2,139 and 179 genes, respectively) were obtained from current literature (Supplementary Table S1).

### Differential expression analysis

Differentially expressed genes (DEGs) [|log2Fold Change (FC)| > 0.5, p < 0.05] were identified between SLE patients and control samples within the training cohort using the limma (v 3.54.0) package (Ritchie et al., 2015). Benjamini–Hochberg adjusted P-values were also calculated and reported to evaluate the false discovery rate. The volcano plot was generated to visualize DEGs using the ggplot2 (v 3.4.4) package (Zhao et al., 2022), with the top 10 up- and down-regulated genes tagged according to their log2FC. The top ten up- and down-regulated DEGs were visualized utilizing a heatmap employing ggplot2 (v 3.4.4) package, sorted by log2FC.

### Identification and enrichment analysis of candidate genes

To investigate the relationship between lactic acid metabolism, NETs and SLE, we used the ggvenn package (v 0.1.9) to examine the intersecting genes of identified DEGs, LAM-RGs and NET-RGs (Zheng et al., 2025). Gene Ontology (GO) and Kyoto Encyclopedia of Genes and Genomes (KEGG) enrichment analyses were performed using clusterProfiler (v 4.7.1.003) (Wu T. et al., 2021) and org.Hs.eg.db (v 3.16.0) (Qing et al., 2022) with a significance threshold of p < 0.05 and FDR <0.05. To complement the over-representation analysis based on the 28 candidate genes, rank-based gene set enrichment analysis (GSEA) was further performed using the full differential expression ranked gene list between SLE and control samples. Genes were ranked according to the differential expression statistic, with positive ranking values corresponding to higher expression in SLE samples. GO biological process, KEGG, and Hallmark gene sets from MSigDB were used as reference gene sets. Normalized enrichment scores (NES), nominal p values, and FDR q values were reported for representative pathways. These candidate-gene enrichment analyses were interpreted as exploratory and were complemented by full-ranked GSEA. Protein-protein interactions (PPIs) for candidate genes were retrieved from the STRING database (http://string-db.org) (confidence ≥0.4) and visualized with Cytoscape (v 3.9.1) (Liu et al., 2020) to construct a PPI network and obtain their protein level interactions (confidence ≥0.4). Degree centrality in Cytoscape was calculated to evaluate the importance of candidate genes.

### Mendelian randomization (MR) analysis

To explore MR-based genetic associations between candidate genes and SLE, GWAS data (ebi-a-GCST003156) from the IEU-Open GWAS Catalog were analyzed, which comprised 14,267 European samples (5,201 SLE cases, 9,066 controls) and 7,071,163 SNPs. eQTLs of candidate genes were retrieved as exposure factors (Supplementary Table S2). Instrumental variables (IVs) were screened by removing those with linkage disequilibrium (p < 5 × 10^−6^, clump = TRUE, r^2^ = 0.001, kb = 100) (Chu et al., 2025; Huang et al., 2024) and weak IVs (F-statistic <10), SNPs with no significant association with the exposure gene were excluded, and only independent, strongly associated instrumental variables were retained, using VariantAnnotation (v 1.44.0) (Obenchain et al., 2014) and ieugwasr (v 0.2.1) (Bourgault et al., 2023). The harmonized data function in TwoSampleMR (v 0.6.1) (Hemani et al., 2018) aligned effect alleles and sizes for exposure-outcome matching, and genes with ≥3 SNPs proceeded to MR analysis.

MR analysis was performed by TwoSampleMR (v 0.6.1) with five algorithms: MR-Egger (Bowden et al., 2015), Weighted median (Bowden et al., 2016), Inverse variance weighted (IVW) (Burgess et al., 2015), Simple mode (Hemani et al., 2018), Weighted mode (Hartwig et al., 2017), the IVW method was prioritized as the primary MR estimation method, while the other four algorithms served for cross-validation of result stability, prioritizing IVW. Odds ratios (OR) > 1 indicated risk factors, while OR < 1 indicated protective factors. OR values and 95% confidence intervals (95% CI) were reported to quantify the MR association estimates. Robustness of results was further assessed by heterogeneity tests, horizontal pleiotropy tests, and leave-one-out tests. Cochran’s Q test for heterogeneity, MR-Egger regression intercept test for horizontal pleiotropy, and leave-one-out analysis to assess individual SNP influence. Potential biomarkers were identified by Steiger filtering for results with steiger dir = TRUE and p < 0.05, Steiger filtering was used to assess the directionality of the genetic association. To control the risk of false positives due to multiple testing, the Benjamini–Hochberg (BH) method (p.adjust, method = “BH”) was used to perform FDR correction on the P values from MR heterogeneity, horizontal pleiotropy, and Steiger tests. Colocalization analysis was further conducted using the coloc package (v 5.2.3) to evaluate the colocalization between gene expression and SLE genetic signals. These MR-related analyses were interpreted as supportive rather than definitive evidence.

### Recognition of biomarkers

Genes that showed significant inter-group differences and consistent expression trends across both training and validation cohorts (p < 0.05) were identified as potential biomarkers, and their performances examined by the receiver operating characteristic (ROC) curves using the pROC (v 1.18.0) package (Robin et al., 2011). Genes with area under curve (AUC) exceeding 0.7 in both cohorts were selected as biomarkers for further investigation.

### Construction of a nomogram through biomarkers

Calibration curves were generated with nomogramEx (v 3.0) (Balachandran et al., 2015) and ResourceSelection (v 0.3.5) (Haze, 2023) to assess predictive performance. The Hosmer-Lemeshow test (p > 0.05) was used to confirm model fit by showing no significant deviation between predicted and actual outcomes. Clinical utility was evaluated through decision curve analysis (DCA) using the plot_decision_curve function in rmda (v 1.6.1) (Vickers et al., 2008). A clinical impact curve (CIC) was also generated with the plot_clinical_impact function in rmda to quantify the nomogram’s net benefit.

### Comprehensive functional characterization analysis

To elucidate the functional characteristics of the potential biomarkers, the “c2. all.v7.2.symbols.gmt” gene set from MSigDB (https://www.gsea-msigdb.org/gsea/msigdb) was used as a reference. Gene set variation analysis (GSVA) scores were quantified using the GSVA package (v 1.46.0) (Hänzelmann et al., 2013), enabling comparison of biological functions between SLE and control samples (p < 0.05, |t| > 2, FDR <0.05). Spearman correlation analysis between the top 10 pathways (ranked by |t| values) and biomarkers was performed with the quickcor function in ggcor (v 0.9.8.1) (Spearman, 2010), with results visualized in a heatmap (|R| > 0.30, p < 0.05).

Gene functions and interactions were predicted using the GeneMANIA plugin in Cytoscape (v 3.9.1), constructing a gene-gene interaction (GGI) network. Organ-specific expression data for biomarkers were retrieved from the GTEx database (https://ngdc.cncb.ac.cn/databasecommons/) to evaluate biomarker expression across tissues.

### Immune cell analysis

ssGSEA algorithm from the GSVA package (v 1.46.0) was used to assess the relative abundance of 28 immune cell types in the peripheral blood of SLE patients and healthy controls (Charoentong et al., 2017). Immune cells with significantly different proportions (p < 0.05) were identified and visualized with box plots using ggplot2 (v 3.4.4). Spearman correlation analyses were conducted with the stats package (v 4.2.2) to explore relationships between potential biomarkers and immune cells with differential proportions (|R| > 0.30, p < 0.05, FDR <0.05). Correlations were visualized as heatmaps using ggcorrplot (v 0.1.4) (Lu et al., 2022) and lollipop diagrams using ggdotchart from ggpubr (v 0.6.0) (Bian et al., 2024). The immune-cell deconvolution and subsequent biomarker–immune cell correlation analyses were interpreted as exploratory.

### Construction of regulatory networks

In order to delve into the molecular regulatory mechanisms for the potential biomarkers, the relationships between transcription factors (TFs) and biomarkers were predicted using the NetworkAnalyst database (https://www.networkanalyst.ca/). Following that, miRNAs targeting the implicated biomarkers were forecasted using the MicroRNA Target Prediction Database (miRDB) (https://mirdb.org/). By leveraging the TFs and miRNAs associated with the biomarkers, the TF-mRNA-miRNA molecular regulatory network was constructed and graphically represented using ggsankey (v 0.0.9) package (Wu Q. et al., 2021).

### ScRNA-seq analysis

The GSE135779 dataset was analyzed using the Seurat package (v 5.0.1) (Yu et al., 2022) to explore the cellular mechanisms underlying SLE. Quality control (QC) was performed to exclude low-quality cells based on three criteria: (Morand et al., 2023): gene counts ranging from 600 to 1,800 (Tsokos, 2020), mitochondrial gene content being ≤5%, and (Siegel and Sammaritano, 2024) total gene expression being ≤6,000. QC results were visualized using VlnPlot. Data normalization was carried out using the NormalizeData function, and 2,000 highly variable genes (HVGs) were identified through FindVariableFeatures. Principal component analysis (PCA) was performed on the HVGs using RunPCA, with the optimal principal components being determined by JackStraw and ElbowPlot methods. Following dimensionality reduction, UMAP was applied to visualize clustering, and cell clusters were identified by using FindNeighbors and FindClusters (resolution = 0.4). The expression of marker genes, identified from previous literature (Tang et al., 2021; Wang et al., 2022), was assessed across clusters using DotPlot and was further analyzed with ggplot2 (v 3.4.4). To reduce the risk of pseudoreplication in group-level comparisons, ITGAM and TKT expression between SLE and control groups within each annotated cell type was assessed using donor-level summaries. For each donor and cell type, normalized expression values were averaged across cells, and the resulting donor-level values were compared between groups. Each donor, rather than each individual cell, was used as the statistical unit.

### Enrichment of cell types and recognition of the key cells

In order to explore the biological functions associated with annotated cells, functional enrichment of annotated cells was carried out using the analyse_sc_clusters function in ReactomeGSA (v 1.12.0) package (Griss et al., 2020). The top 10 pathways were presented by a heatmap generated using the plot_gsva_heatmap function from ReactomeGSA (v 1.12.0) package.

Further analysis included observing the abundance of each cell cluster between SLE and control samples and delineating the expression of biomarkers in each cell cluster within GSE135779. Group-level comparisons of ITGAM and TKT expression within each annotated cell type were then performed using donor-level summaries, with each donor treated as the biological replicate. Based on the altered cell-type composition observed in SLE samples, T cells were selected for subsequent exploratory pseudo-time and cell-cell communication analyses. To investigate the differentiation trajectory and evolution of key cells during development, the monocle (v 2.26.0) package (Trapnell et al., 2014) was adopted to conduct pseudo-time series analysis, focusing on the biomarkers expression changes over time in the key cells.

### Analysis of key cells communication

To further understand the molecular relationships and interactions between different cell types, CellChat package (v 1.6.1) (Jin et al., 2021) was engaged in communication analysis within the annotated cell types. The study also presented a comprehensive visualization of receptor-ligand interplays through a bubble chart, depicting the exchange of signals received and dispatched by the annotated cell types.

### Animals and LN model establishment

Female C57BL/6 mice (6–8 weeks old) were acquired from the Guangzhou Yuanjun Biotechnology Co., Ltd. (Guangzhou, China). Female mice were selected because SLE and LN show a strong female predominance, and the use of a single sex helped reduce sex-related biological variability in this *in vivo* validation experiment. In this study, we did not use classical spontaneous LN models (e.g., MRL/lpr or NZB/W F1) because their lupus diseases are genetically driven and show variable disease onset and background-dependent immune phenotypes (Davidson and Aranow, 2010; Huston and Steinberg, 1979). Here the apoptotic cell-induced model provides an inducible experimental SLE/LN-like system based on repeated exposure to apoptotic autoantigens, systemic autoimmune activation, anti-dsDNA antibody production, and renal inflammatory injury (Li et al., 2015). This model also provides a relatively controllable experimental window, which allowed us to examine TKT-associated metabolic remodeling, immune-cell infiltration, NET-associated inflammatory activity, and renal injury under the same experimental setting. All animal care and experimental procedures were strictly conducted under the approval of the Animal Ethics Committee of Southern Medical University. The SLE/LN-like model was induced via the intravenous delivery of syngeneic apoptotic cells. Specifically, mice were injected with 1 × 10^7 UV-irradiated apoptotic thymocytes through the tail vein once weekly for four consecutive weeks. Disease progression was allowed to develop naturally for 3–4 months.

Upon phenotypic confirmation of LN, both the wild-type (WT) and LN cohorts were further subdivided into untreated and treated groups. Mice were randomly divided into four groups: WT, WT + oxythiamine, LN, and LN + oxythiamine (n = 5 mice per group). The untreated WT and LN mice were maintained without any interventions. In contrast, the treated WT and LN groups received continuous daily intraperitoneal injections of oxythiamine (HY-107430; MedChemExpress) at a dosage of 100 mg/kg/day for 14 days. The schematic outline of the *in vivo* protocol is detailed in Figure 12A. At the experimental endpoint, all animals were euthanized for subsequent tissue and serum collection.

### Flow cytometry analysis of cell apoptosis

To strictly verify the apoptosis rate of the UV-irradiated thymocytes prior to *in vivo* intravenous injection, flow cytometry was performed using the BD Pharmingen™ FITC Annexin V Apoptosis Detection Kit I (Cat. No. 556547; BD Biosciences, San Jose, CA, United States) according to the manufacturer’s protocols. Briefly, following UV irradiation and the subsequent 3-h incubation, primary thymocytes were harvested, washed twice with cold phosphate-buffered saline (PBS), and resuspended in 1× Binding Buffer. Subsequently, the cell suspension was stained with Annexin V-FITC and Propidium Iodide (PI) for 15 min at room temperature in the dark. The apoptosis rate (comprising both early and late apoptotic cells) was immediately analyzed using a BD LSRFortessa™ cell analyzer (BD Biosciences). The flow cytometry data were quantified and analyzed utilizing FlowJo software (Tree Star).

### Serum creatinine and BUN measurement

The levels of serum creatinine and BUN were measured using an AU480 chemistry analyzer (Beckman Coulter, Atlanta, GA, United States).

### Urinary protein-to-creatinine ratio measurement

Urine samples were collected at the experimental endpoint. Urinary protein concentration was measured using a urine protein test kit (C035-2-1; Nanjing Jiancheng Bioengineering Institute, Nanjing, China), and urinary creatinine concentration was measured using the Cayman Urinary Creatinine Kit (500701; Cayman Chemical, Ann Arbor, MI, United States), according to the manufacturers’ protocols. The urinary protein-to-creatinine ratio (UPCR) was calculated to assess proteinuria.

### Lactate measurement

Mouse Serum Lactate Assay Kit was purchased from Abcam (ab65331, United Kingdom). Serum lactate was measured according to the procedures specified by the manufacturer.

### Elisa

Mouse anti-dsDNA antibody ELISA Kit was purchased from Cusabio (CSB-E11194m, China). Serum anti-dsDNA antibody was measured according to the procedures specified by the manufacturer.

### MPO-DNA complex measurement

MPO-DNA complexes were measured in mouse plasma using a capture ELISA-based method. Briefly, plasma samples were collected at the experimental endpoint and stored at −80 °C until analysis. MPO-containing complexes were captured using a mouse MPO ELISA kit (Hycult Biotech, HYC-HK210-01). After incubation with diluted plasma samples and repeated washing, the DNA component of the captured MPO-containing complexes was detected using the peroxidase-labeled anti-DNA antibody from the Cell Death Detection ELISAPLUS kit (Roche Diagnostics GmbH, Cat. No. 11774425001). Substrate solution was then added for color development, and absorbance was measured using a microplate reader. MPO-DNA complex levels were expressed as relative absorbance values and normalized to the mean value of the WT group.

### Renal histopathology, immunohistochemistry, and immunofluorescence analysis

Kidney tissues were fixed in 4% paraformaldehyde for 24 h, embedded in paraffin, and cut into 3-μm sections. To evaluate macroscopic renal damage and interstitial fibrosis, sections were stained with Hematoxylin and Eosin (H&E), Periodic Acid-Schiff (PAS), and Masson’s trichrome following standard protocols.

For immunostaining, the 3-μm sections were deparaffinized, rehydrated, and subjected to heat-induced antigen retrieval in sodium citrate buffer (pH 6.0). After blocking with 5% BSA for 1 h at room temperature, sections were incubated overnight at 4 °C with specific primary antibodies. For immunohistochemistry, PCNA (sc-71858, Santa Cruz) expression was visualized using an HRP-conjugated secondary antibody and 3,3′-diaminobenzidine (DAB) substrate.

For immunofluorescence, sections were incubated with the following primary antibodies: CD14 (MA1-40064, Thermo Fisher), TKT (11039-1-AP, Proteintech), ITGAM (A1581, ABclonal), CD3 (ab11089, Abcam), F4/80 (A27257, ABclonal), Ly6G (GB11229-100, Servicebio), Fn (A12977, ABclonal), α-SMA (A7248, ABclonal), and CPT1A (A5307, ABclonal). For TKT/CD14 co-localization staining, sections were incubated with a primary antibody combination from distinct host species. After washing with PBS, sections were incubated with the corresponding Cy3-or Cy2-conjugated secondary antibodies (Jackson ImmunoResearch Laboratories) for 1 h at room temperature in the dark.

For renal IgG and C3 deposition analysis, frozen kidney sections were used for immunofluorescence staining. Briefly, OCT-embedded renal tissues were cut into 5-μm sections, fixed with cold acetone for 10 min, and washed with PBS. After blocking with 5% BSA for 1 h at room temperature, sections were incubated with ABflo® 594-conjugated goat anti-mouse IgG (H + L) antibody (AS054; ABclonal) to detect renal IgG deposition. For C3 staining, sections were incubated with anti-C3 antibody (A6879; ABclonal) overnight at 4 °C, followed by incubation with the corresponding fluorescence-conjugated anti-rabbit secondary antibody for 1 h at room temperature in the dark.

For Cit-H3/Ly6G co-localization staining, TSA-based multiplex immunofluorescence staining was performed. Sections were sequentially incubated with anti-Cit-H3 antibody (Histone H3 citrulline R3, ab219407, Abcam) and anti-Ly6G antibody [EPR22909-135] (ab238132, Abcam). After each primary antibody incubation and PBS wash, sections were incubated with HRP-conjugated secondary antibody, followed by signal amplification using TSA-480 (TSA-02, Ruisaiqi Biotechnology) and TSA-570 (TSA-04, Ruisaiqi Biotechnology). Heat-mediated stripping was performed between staining rounds. Nuclei were counterstained with DAPI (Sigma-Aldrich) for 10 min. Fluorescence images were acquired using a Nikon A1R confocal microscope (Nikon Corporation, Tokyo, Japan) and quantified using ImageJ software.

### Western blotting analysis

Renal tissues were homogenized in lysis buffer, and protein concentrations were determined utilizing a BCA protein assay. Equal amounts of total protein were resolved by SDS-PAGE and electrotransferred onto PVDF membranes (Merck Millipore). Membranes were blocked with 1% bovine serum albumin for 1 h at room temperature, followed by overnight incubation at 4 °C with the following primary antibodies: anti-ITGAM (A1581; ABclonal), anti-TKT (11039-1-AP; Proteintech), anti-fibronectin (A12977; ABclonal), anti-α-SMA (A7248; ABclonal), anti-Cit-H3 (ab5103; Abcam), anti-GAPDH (AC033; ABclonal), and anti-α-tubulin (AC012; ABclonal). Membranes were subsequently incubated with HRP-conjugated secondary antibodies for 1 h at room temperature. Protein bands were visualized using an ECL detection system (Applygen, Beijing, China) and quantified via densitometry.

### RNA extraction and quantitative real-time PCR

Total RNA was isolated from renal tissues and reverse-transcribed into cDNA utilizing respective commercial kits (Vazyme, Nanjing, China) according to the manufacturer’s protocols. Quantitative real-time PCR was conducted on a LightCycler 480 II system (Roche Diagnostics, Basel, Switzerland) using the SYBR Green qPCR Master Mix (Vazyme). Relative mRNA expression levels of target genes (*Itgam*, *Tkt*, *Il6*, *Tnfa*, and *Il1b*) were calculated via the 2^−ΔΔCt^ method, with Actb (β-actin) serving as the endogenous normalization control. All specific primer sequences are detailed in Supplementary Table S3.

### Statistical analysis

Statistical evaluations were conducted using GraphPad Prism 9.0 and R (v4.2.2). Data normality was first assessed via the Shapiro-Wilk test. For two-group comparisons, we employed Student’s t-test for normally distributed parameters or the Wilcoxon rank-sum test for non-parametric data. In experiments involving multiple groups, statistical significance was determined using one-way ANOVA followed by Tukey’s *post hoc* test, or the Kruskal-Wallis test with Dunn’s multiple comparisons for non-normal datasets. Spearman’s rank correlation was used to evaluate associations between variables. Data are expressed as mean ± SEM (or SD). A two-tailed P < 0.05 was defined as the threshold for significance.

## Results

### Enrichment analysis and PPI network of 28 candidate genes

**FIGURE 1:**
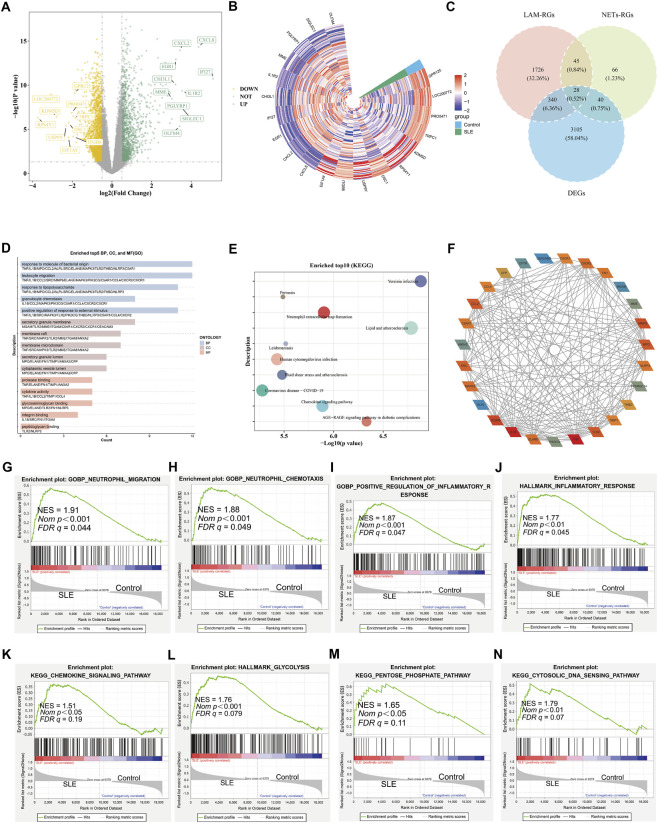
Enrichment analysis and PPI network of 28 candidate genes. **(A,B)** Volcano plots illustrated the differentially expressed genes (DEGs) identified between SLE and control samples, with 1,428 up-regulated and 2,085 down-regulated genes (p < 0.05). **(C)** Venn diagram showed the intersection of 3,513 DEGs, 2,139 LAM-RGs, and 179 NETs-RGs, resulting in 28 candidate genes. **(D)** Gene Ontology (GO) enrichment analysis was performed on the 28 candidate genes, showed representative BP (biological process) terms such as “response to molecule of bacterial origin”, “leukocyte migration”, and “positive regulation of response to external stimulus”; CC (cellular component) terms like “secretory granule membrane” and “membrane raft”; and MF (molecular function) terms including “protease binding” and “cytokine activity” were obtained (p < 0.05 and FDR <0.05). **(E)** KEGG pathway enrichment analysis identified pathways such as “*yersinia* infection”, “lipid and atherosclerosis”, and “neutrophil extracellular trap formation” (p < 0.05 and FDR <0.05). **(F)** Protein-protein interaction (PPI) network was constructed based on the 28 candidate genes, which showed 27 interconnected genes and 1 discrete gene (TKT). ITGAM, IL1B, and TNF were identified as the top 3 hub genes with the highest degree scores. **(G–N)** To complement the over-representation analysis based on the 28 candidate genes, representative GSEA plots were generated using the full differential expression ranked gene list between SLE and control samples. Selected pathways related to neutrophil recruitment, inflammatory responses, immune signaling, and metabolic remodeling showed positive enrichment in SLE samples, including neutrophil migration, neutrophil chemotaxis, positive regulation of inflammatory response, inflammatory response, chemokine signaling pathway, glycolysis, pentose phosphate pathway, and cytosolic DNA-sensing pathway. The normalized enrichment score (NES), nominal p value, and FDR q value are indicated in each panel.

Following differential expression analysis in the training cohort between SLE and control samples, 3,513 DEGs were identified (1,428 being up-regulated, 2,085 being down-regulated, p < 0.05) (Figures 1A,B, Supplementary Table S4). An intersection of 3,513 DEGs, 2,139 LAM-RGs, and 179 NETs-RGs revealed 28 candidate genes (Figure 1C, Supplementary Table S4). GO analysis identified 1,116 BP terms (such as, “response to molecule of bacterial origin”, “leukocyte migration”), 55 CC terms (e.g., “secretory granule membrane”, “membrane raft”), and 91 MF terms (e.g., “protease binding”, “cytokine activity”) (p < 0.05 and FDR <0.05) (Figure 1D, Supplementary Table S4). Additionally, 84 KEGG pathways were enriched, including “*yersinia* infection” and “neutrophil extracellular trap formation” (p < 0.05 and FDR <0.05) (Figure 1E, Supplementary Table S4). A PPI network based on 27 candidate genes identified ITGAM, IL1B, and TNF as top-ranked by Degree, while TKT being a discrete gene (Figure 1F, Supplementary Table S5). These findings underlined the functional relevance of candidate genes in SLE progression. To further determine whether the pathways implicated by the 28 candidate genes were supported by coordinated transcriptomic alterations, we performed rank-based GSEA using the full differential expression ranked gene list. In line with the enrichment results from the candidate gene set, neutrophil- and inflammation-related pathways were positively enriched in SLE samples, including neutrophil migration, neutrophil chemotaxis, positive regulation of inflammatory response, and inflammatory response (Figures 1G–J). Notably, neutrophil chemotaxis remained significant after FDR correction (NES = 1.88, nominal p < 0.001, FDR q = 0.049). Immune signaling and metabolism-related pathways, including chemokine signaling, glycolysis, pentose phosphate pathway, and cytosolic DNA-sensing pathway, also showed positive enrichment in SLE samples (Figures 1K–N).

### Recognition of three potential biomarkers with genetic correlations to SLE

**FIGURE 2:**
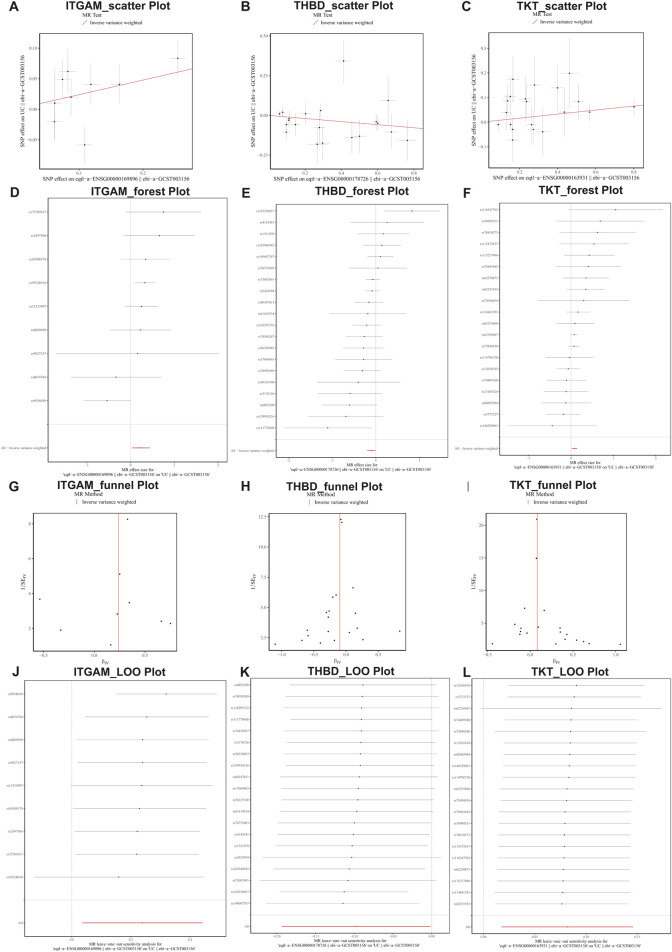
Three potential biomarkers with genetic correlations to SLE were identified through Mendelian randomization (MR) analysis **(A–C)** Scatter plots of the inverse-variance weighted (IVW) method demonstrated the genetic correlations of THBD, ITGAM, and TKT with SLE, where the fold and intercepts of the vertical coordinates were near zero, indicating minimal confounding effects. **(D–F)** Forest plots displayed the effect size of the three genes: ITGAM (OR = 1.270, 95% CI = 1.036–1.557, p = 0.021) and TKT (OR = 1.085, 95% CI = 1.018–1.157, p = 0.013) as risk factors, and THBD (OR = 0.907, 95% CI = 0.824–0.999, p = 0.047) as a protective factor for SLE. **(G–I)** The uniform distribution of the genetic loci on both sides of the midline aligned with Mendel’s second law, further supporting the robustness of the results. **(J–L)** Leave-one-out (LOO) sensitivity analysis verified the robustness and reliability of the MR analysis, showing that the genetic correlations of THBD, ITGAM, and TKT with SLE remained consistent.

After screening, 23 candidate genes were retained for MR analysis (Supplementary Table S6). MR identified 3 genes (THBD, ITGAM, TKT; SNP ≥3) that were associated with SLE (p < 0.05, IVW) (Supplementary Table S7). ITGAM (OR 1.270, 95% confidence interval (CI) 1.036–1.557, p = 0.021) and TKT (OR 1.085, 95% CI 1.018–1.157, p = 0.013) were associated with increased risk of SLE, while THBD (OR 0.907, 95% CI 0.824–0.999, p = 0.047) showed reduced risk. Scatter plots showed generally consistent MR estimates across different methods (Figures 2A–C). Forest plots showed ITGAM and TKT loci as risk factors and THBD as protective factor for SLE (Figures 2D–F), with gene distribution being consistent with Mendel’s second law (Figures 2G–I). Sensitivity analyses confirmed no heterogeneity (p > 0.05, Supplementary Table S8) or confounders (p > 0.05, Supplementary Table S9). The LOO test demonstrated the robustness of MR results (Figures 2J–L), and the Steiger test supported the directionality of the genetic association (steiger dir = TRUE, p < 0.05, Supplementary Table S10). The FDR-corrected results of the MR heterogeneity test, horizontal pleiotropy test, and Steiger test are presented in Supplementary Tables S11-S13. Following FDR correction via the BH method, the results of MR sensitivity analyses remained reliable. Further colocalization analysis revealed that all PPH4 values for TKT and ITGAM with SLE (ebi-a-GCST003156) were less than 0.6, indicating no clear evidence of shared causal variants between the two genes and SLE, with insufficient overlap in genetic pathogenic loci.

### Identification and validation of ITGAM and TKT in SLE cohorts and human LN renal tissue

**FIGURE 3:**
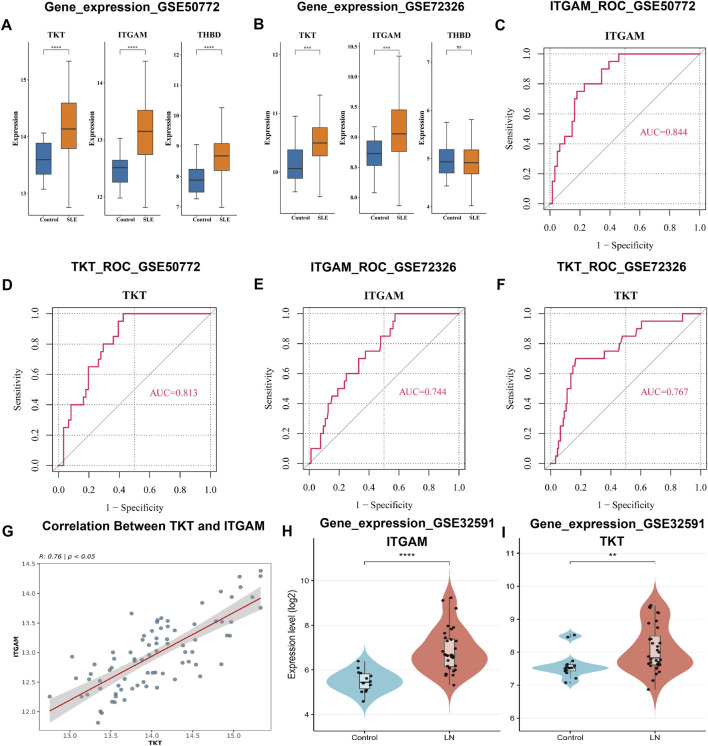
Validation of ITGAM and TKT in peripheral blood SLE cohorts and human LN renal biopsy tissue. **(A,B)** Boxplots showed significant upregulation of ITGAM and TKT in SLE samples compared to control samples across both the training and validation cohorts. **(C–F)** Receiver operating characteristic (ROC) curves illustrated the robust diagnostic performance of ITGAM and TKT, with AUC values exceeding 0.7 in both cohorts, highlighting their capacity to distinguish SLE from control samples **(G)** Scatter plot showed a significant positive correlation between ITGAM and TKT expression levels (R = 0.76, p < 0.05). **(H,I)** Violin plots with embedded boxplots showing the expression levels of ITGAM and TKT in control glomerular samples (n = 14) and LN glomerular samples (n = 32) from the human LN renal biopsy dataset GSE32591. ITGAM and TKT were both significantly increased in LN glomerular samples compared with controls, further supporting their relevance to renal involvement in LN. Statistical significance is indicated as follows: ns, not significant; **p < 0.01; ***p < 0.001; and ****p < 0.0001.

After identifying MR-based associations between THBD, ITGAM, TKT and SLE, expression analysis revealed significant differences in ITGAM and TKT between SLE and control samples in both the training and validation cohorts, with both genes being uniformly up-regulated in SLE (p < 0.05) compared to control samples (Figures 3A,B). In the training and validation cohorts, ITGAM and TKT both showed AUC values >0.7, further demonstrating their performance to differentiate between SLE and control samples (Figures 3C–F). There was also a significant positive association between ITGAM and TKT (R = 0.76, p < 0.05) (Figure 3G). Consequently, ITGAM and TKT were selected as potential biomarkers for predicting development of SLE. To further assess the renal relevance of these candidate genes at a tissue level in human LN, we analyzed the glomerular compartment of the human LN renal biopsy dataset GSE32591. Both ITGAM and TKT were significantly increased in LN glomerular samples compared with control glomerular samples (Figures 3H,I), suggesting that these genes are also dysregulated in human LN renal tissue.

### Developing a nomogram with robust predictive performance for SLE

**FIGURE 4:**
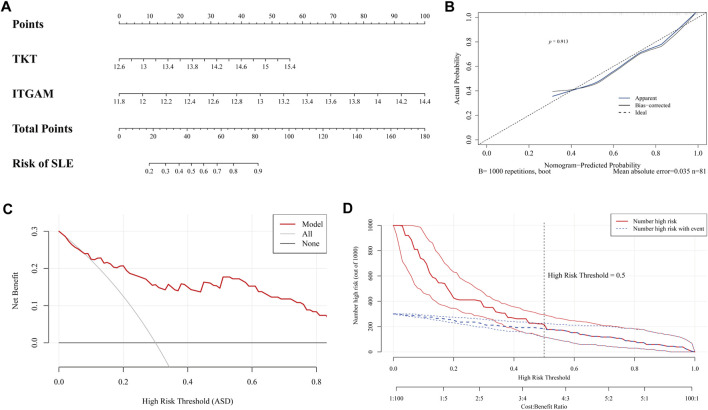
Development and evaluation of a nomogram for predicting the risk of SLE. **(A)** The nomogram was constructed to quantify each biomarker’s contribution and predict the risk of SLE in the training cohort. **(B)** The calibration curve, evaluated by the Hosmer-Lemeshow (HL) test (p = 0.913), demonstrated excellent agreement between predicted and observed outcomes, indicating a good model fit. **(C)** Decision curve analysis (DCA) revealed the superior net benefit of the nomogram across a range of threshold probabilities, supporting its clinical utility. **(D)** The clinical impact curve (CIC) further highlighted the nomogram’s satisfactory clinical applicability and predictive performance.

Based on the above data, a nomogram was constructed for the training cohort, translating each biomarker’s contribution into a score to maximally assess the risk of SLE (Figure 4A). The calibration curve, assessed by the Hosmer-Lemeshow (HL) test suggested an excellent model fit (p = 0.913) and confirmed the nomogram’s precision in predictive performance (Figure 4B). In addition, decision curve analysis (DCA) indicated that the nomogram provided a superior net benefit (Figure 4C), while the clinical impact curve (CIC) also highlighted the nomogram’s satisfactory clinical utility (Figure 4D).

### Functional analysis of candidate genes

**FIGURE 5:**
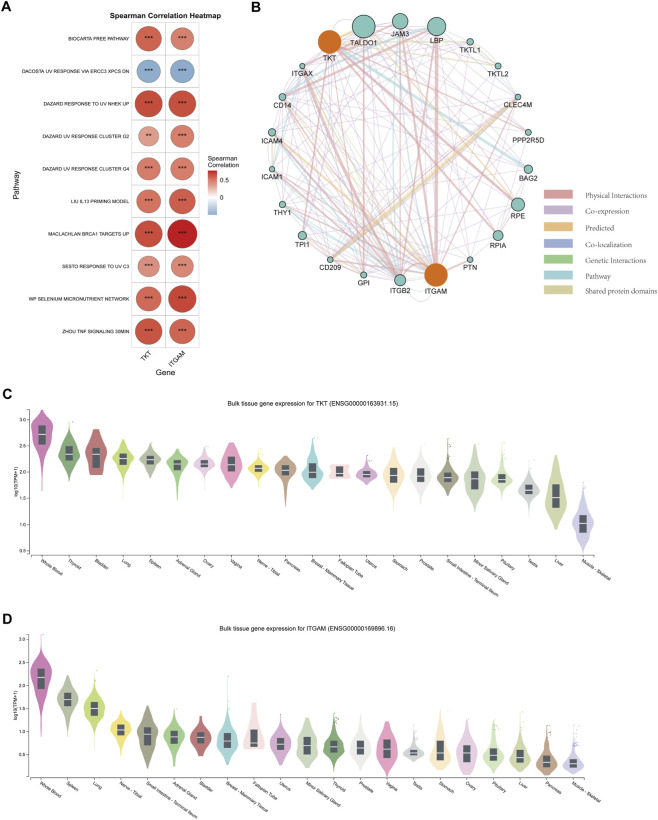
Functional analysis and expression patterns of biomarkers. **(A)** Gene Set Variation Analysis (GSVA) identified 3,176 pathways with significant differences between SLE and control groups (|t| > 2, p < 0.05). ITGAM and TKT showed the most significant correlations with pathways, including negative associations with “DaCosta UV response via ERCC3 XPCS DN” and positive associations with “MacLachlan BRCA1 targets UP” and “Dazard response to UV NHEK UP” (p < 0.05). **(B)** GeneMANIA co-expression network analysis revealed 20 genes related to the biomarkers involved in physical and genetic interactions (e.g., TALDO1, JAM3, and LBP). **(C,D)** GTEx database analysis showed that ITGAM and TKT are predominantly expressed in whole blood, indicating their relevance to blood-related processes.

GSVA revealed 3,176 pathways with significant differences between SLE and control groups (|t| > 2, p < 0.05 and FDR <0.05) (Supplementary Table S14). Correlation analysis showed that biomarkers were significantly associated with the top 10 pathways (Figure 5A). ITGAM and TKT had the strongest negative correlations with “DaCosta UV response via ERCC3 XPCS DN” (R = −0.500 and −0.489, p < 0.05), while ITGAM and TKT showed positive correlations with “MacLachlan BRCA1 targets UP” (R = 0.771, p < 0.05) and “Dazard response to UV NHEK UP” (R = 0.664, p < 0.05), respectively, reflecting key biological functions in SLE.

GeneMANIA co-expression network analysis suggested potential molecular interactions with 20 genes related to the biomarkers, including TALDO1, JAM3, and LBP, which may participate in “physical interactions”, “genetic interactions”, and “shared protein domains” (Figure 5B). GTEx database analysis indicated that TKT and ITGAM tend to be highly expressed in whole blood, implying potential roles in blood-related processes (Figures 5C,D).

### Revealing specific immune cell types in SLE

**FIGURE 6:**
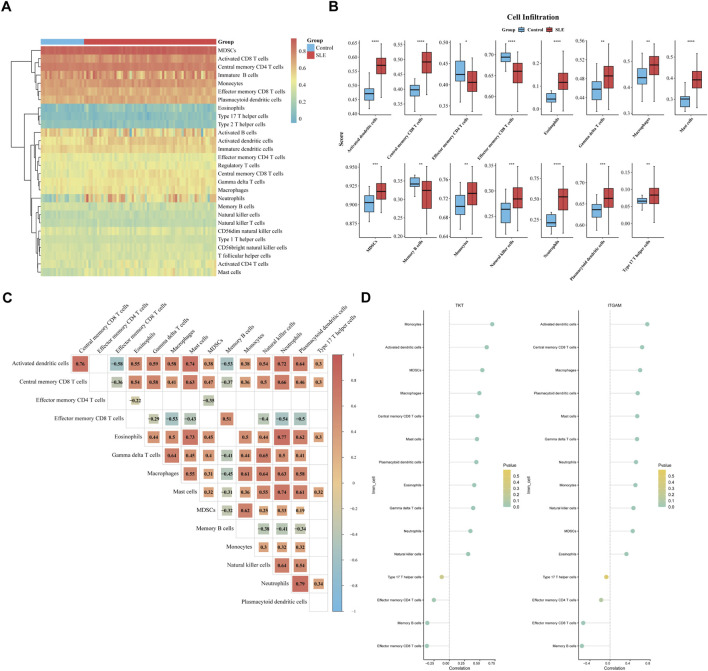
Immune cell analysis in SLE and control samples. **(A)** The ssGSEA scores visualized the proportions of 28 immune cell types in the peripheral blood from SLE patients and healthy controls. **(B)** Boxplots identified significant differences in 15 immune cell types, including activated dendritic cells, effector memory CD4 T cells, memory B cells, eosinophils, and neutrophils (p < 0.05). **(C)** Correlation analysis revealed significant interactions among immune cells present differentially. **(D)** ITGAM and TKT showed significant correlations with immune cells. ITGAM was positively associated with activated dendritic cells (R = 0.742) and negatively with memory B cells (R = −0.562), while TKT was positively correlated with monocytes (R = 0.704) and negatively with effector memory CD8 T cells (R = −0.367) (p < 0.05).

The proportion of 28 immune cells in PBMCs from SLE and control samples were analyzed using ssGSEA scores (Figure 6A). Significant differences in proportions were observed in 15 immune cell types, including activated dendritic cells, central memory CD8 T cells, effector memory CD4 and CD8 T cells, eosinophils, gamma delta T cells, macrophages, mast cells, MDSCs, memory B cells, monocytes, NK cells, neutrophils, plasmacytoid dendritic cells, and Th17 cells (p < 0.05) (Figure 6B).

There were correlations between immune cells that showed significantly different proportions in SLE samples (|R| > 0.30, p < 0.05 and FDR <0.05), including a strong negative relationship between effector memory CD8 T cells and activated dendritic cells (R = −0.576, p < 0.05), and a strong positive correlation between neutrophils and plasmacytoid dendritic cells (R = 0.795, p < 0.05) (Figure 6C, Supplementary Table S15). ITGAM expression correlated positively with activated dendritic cells (R = 0.742, p < 0.05) but negatively with memory B cells (R = −0.562, p < 0.05). TKT expression showed the strongest positive correlation with monocytes (R = 0.704, p < 0.05) but had the strongest negative correlation with effector memory CD8 T cells (R = −0.367, p < 0.05) (Figure 6D, Supplementary Table S15).

### Unraveling regulatory networks of biomarkers

**FIGURE 7:**
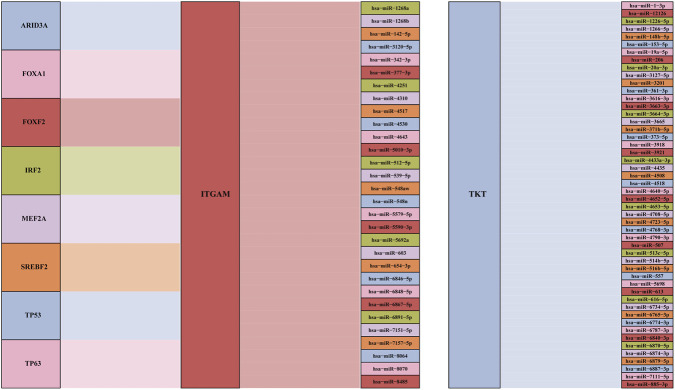
Regulatory networks of ITGAM and TKT in SLE. Transcription factors (TFs) and microRNAs (miRNAs) predicted to regulate ITGAM and TKT were identified using the NetworkAnalyst database. Eight TFs were predicted for ITGAM, while no TFs were identified for TKT. A total of 30 miRNAs were predicted to regulate ITGAM, and 50 miRNAs were associated with TKT. TF-mRNA-miRNA regulatory networks were constructed for each biomarker, highlighting interactions such as hsa-miR-142-5p–ITGAM–TP53 and hsa-miR-1-3p–TKT, providing insights into the potential regulatory mechanisms underlying SLE pathogenesis.

The NetworkAnalyst database was employed to predict TFs that regulated the biomarkers, identifying 8 TFs for ITGAM and none for TKT. Furthermore, a total of 30 miRNAs were predicted for ITGAM, while 50 miRNAs were identified to be related to TKT. The TF-mRNA-miRNA regulatory networks were developed for each biomarker, respectively, with the interactions including hsa-miR-142-5p-ITGAM-TP53 and hsa-miR-1-3p-TKT (Figure 7, Supplementary Table S16).

### scRNA-seq analysis of immune cell types in SLE

**FIGURE 8:**
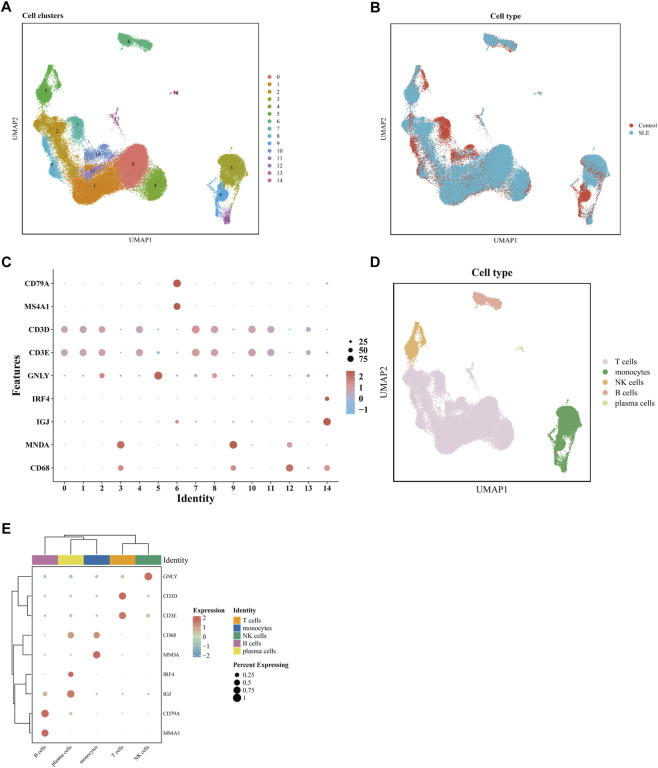
Cell type annotations from scRNA-seq analysis. **(A,B)** UMAP plots showed the clustering of single cells into 15 distinct clusters and their distribution in the GSE135779 dataset. **(C,D)** UMAP plots illustrated the annotation of 15 clusters into five major cell types: monocytes, plasma cells, NK cells, T cells, and B cells, with the proportional representation of each cell type. **(E)** Bubble plot showed the specificity of marker genes, confirming the accuracy of cell type annotations.

scRNA-seq analysis of the GSE135779 dataset was conducted to explore SLE mechanisms at the cellular level. Visualizations of nFeature RNA, nCount RNA, and mitochondrial content before and after QC were generated (Supplementary Figures S1A,B). After processing, 2,000 HVGs were identified, with the top 10 genes showing the most variation highlighted (Supplementary Figure S1C). PCA identified the top 30 PCs for downstream analysis (Supplementary Figure S1D-F). Clustering analysis revealed 15 distinct cell clusters, annotated as monocytes, plasma cells, NK cells, T cells, and B cells (Figures 8A–D). A bubble plot confirmed marker gene specificity, validating the annotations (Figure 8E). To comprehensively profile the functional landscape of these identified populations, we evaluated their global pathway enrichment (Supplementary Figure S2). While fundamental homeostatic and stress-response pathways were broadly represented across these lineages, our subsequent analyses specifically narrowed down on the metabolic and inflammatory perturbations driving SLE pathogenesis.

### Donor-level scRNA-seq analysis of ITGAM and TKT expression in SLE

**FIGURE 9:**
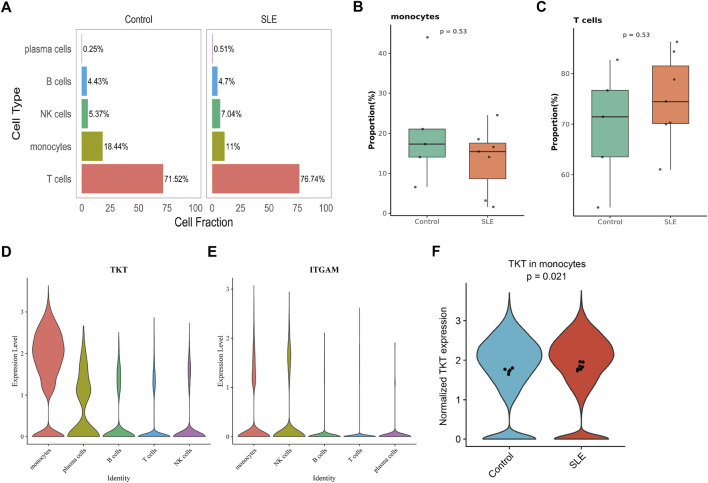
Relationship between ITGAM, TKT and T cells in SLE. **(A)** A histogram showed the proportions of the five cell types in SLE and control samples. **(B,C)** Box plots showed that SLE samples had increased proportions of T cells but lower frequency of monocytes compared to control samples. **(D,E)** Expression levels of ITGAM and TKT in the five cell types, with the highest expression observed in monocytes. **(F)** Donor-level comparison of TKT expression in monocytes between control and SLE samples. Each dot represents one individual donor. TKT expression was higher in monocytes from SLE patients than in controls (p = 0.021).

Following the enrichment analysis, 5 immune cell types were selected to evaluate the proportion in the different samples. Histograms displayed the proportions of these 5 immune cell types in SLE and control samples (Figure 9A), and SLE samples exhibited increased proportions of T cells but lower frequency of monocytes compared to control samples. (Figures 9B,C). Both ITGAM and TKT were expressed in T cells and monocytes, with the highest expression in monocytes (Figures 9D,E, Supplementary Figure S3). To avoid using individual cells as independent replicates, we further summarized ITGAM and TKT expression at the donor level within each annotated cell type. TKT expression was higher in monocytes from SLE patients than in controls (p = 0.021), whereas the other donor-level comparisons for ITGAM and TKT did not reach statistical significance (Figure 9F). These scRNA-seq results were therefore interpreted as exploratory, with the clearest donor-level difference observed for TKT expression in monocytes.

### Pseudo-time trajectory inference and specific communications of key cells

**FIGURE 10:**
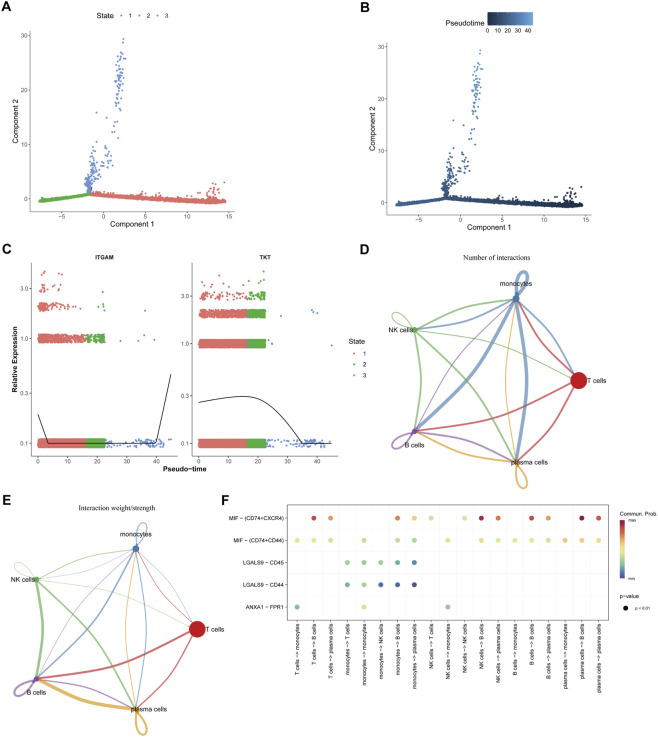
Pseudo-time trajectory and communication patterns of T cells. **(A)** Pseudo-time trajectory analysis categorized T cells into three differentiation stages. **(B)** A temporal trajectory showed the maturation of T cells from the inferred starting point **(C)** Dynamic expression patterns of ITGAM and TKT along the pseudo-time trajectory, reflected stage-specific biological functions. **(D,E)** Cell-cell communication networks highlighted interactions between T cells and other cell types, with particularly strong connections between T cells and B cells. **(F)** Bubble plots showed receptor-ligand interactions, including LGALS9-CD45 and LGALS9-CD44 as signals to T cells, and MIF-related pairs (MIF-CD74+CXCR4, MIF-CD74^+^CD44) as signals from T cells to other cell types. This figure illustrated the differentiation trajectory of T cells and their communication networks, providing insights into their roles and interactions.

Cell trajectory analysis mapped T cells into 3 cellular states (Figures 10A,B), which were then mapped against the ITGAM and TKT expression patterns (Figure 10C). ITGAM expression in SLE T cells shows an initial drop during early differentiation stages, which then remains stable and starts to increase in later maturation stage. TKT tends to remain high during the first two phases but shows a sharp decline in the later stage. Cell communication analysis revealed interactions between T cells and B cells, monocytes, and plasma cells, with the strongest interactions between T cells and B cells (Figures 10D,E). Bubble plots identified receptor-ligand pairs, such as LGALS9-CD45 and LGALS9-CD44 (from other cells to T cells) and MIF-(CD74+CXCR4) and MIF-(CD74^+^CD44) (from T cells to other cells) (Figure 10F).

### Systemic autoimmunity and lupus nephritis-like renal injury driven by apoptotic cell induction *in vivo*

**FIGURE 11:**
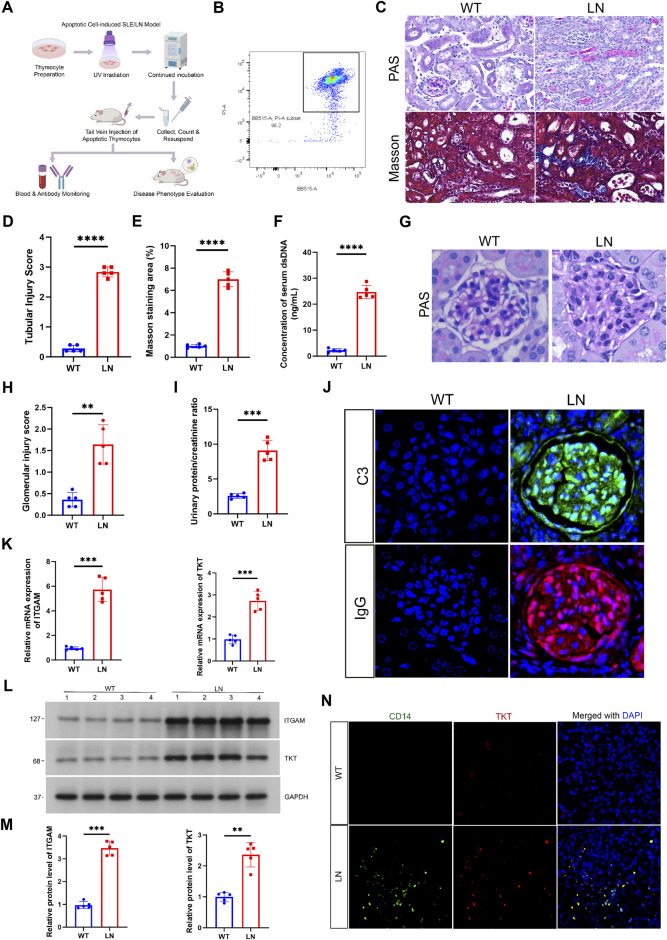
Characterization of renal injury and upregulation of ITGAM and TKT in the apoptotic cell-induced lupus nephritis (LN) model. **(A)** Experimental workflow for the generation of the apoptotic cell-induced LN mouse model. **(B)** Flow cytometry analysis confirming the induction of apoptosis in UV-irradiated thymocytes prior to injection. **(C–E)** Representative PAS and Masson’s trichrome staining of renal sections, with corresponding quantification of tubular injury scores and Masson-positive area, showing tubular damage and interstitial fibrosis in LN mice. **(F)** Serum anti-dsDNA antibody levels, showing increased levels in the LN cohort relative to WT controls **(G,H)** Representative higher-magnification PAS-stained glomeruli and corresponding semi-quantitative glomerular injury scores, showing glomerular involvement in LN mice. **(I)** Urinary protein/creatinine ratio, showing increased proteinuria in LN mice. **(J)** Representative immunofluorescence staining showing renal C3 and IgG deposition in LN mice. **(K)** RT-qPCR analysis showing increased Itgam and Tkt transcript levels in LN mice. **(L,M)** Representative immunoblotting and densitometric quantification showing increased ITGAM and TKT protein levels in LN renal tissues. **(N)** Immunofluorescence staining showing TKT expression (red) in CD14^+^ monocyte-lineage cells (green) in LN renal tissues. Nuclei were counterstained with DAPI (blue). Data are presented as mean ± SD. n = 5 mice per group. **P < 0.01, ***P < 0.001, ****P < 0.0001 compared with the WT group (Student’s t-test).

We established a murine LN model by repeated intravenous injection of UV-irradiated apoptotic thymocytes (Figure 11A). Before injection, apoptosis of the UV-irradiated thymocytes was confirmed by flow cytometry (Figure 11B). Compared with WT control mice, LN mice showed more prominent renal histopathological changes, including tubular injury and interstitial fibrosis, as shown by PAS and Masson’s trichrome staining, tubular injury scoring, and Masson-positive area quantification (Figures 11C–E). LN mice also showed elevated serum anti-dsDNA antibody levels (Figure 11F). Higher-magnification PAS images further showed glomerular involvement, with increased glomerular injury scores in LN mice (Figures 11G,H). In addition, urinary protein/creatinine ratio was increased in LN mice, indicating proteinuria (Figure 11I). Renal immunofluorescence staining showed increased C3 and IgG deposition in LN mice (Figure 11J).

In line with the computational analysis, renal Itgam and Tkt mRNA levels were increased in LN mice (Figure 11K). Western blotting further showed higher ITGAM and TKT protein expression in LN renal tissues (Figures 11L,M). Co-immunofluorescence staining showed TKT expression in CD14^+^ monocyte-lineage cells in LN kidneys (Figure 11N). Together, these findings indicate that apoptotic cell induction led to systemic autoimmunity with lupus nephritis-like renal injury, accompanied by increased renal ITGAM and TKT expression.

### Therapeutic inhibition of TKT attenuates renal injury and inflammatory signaling in LN mice

**FIGURE 12:**
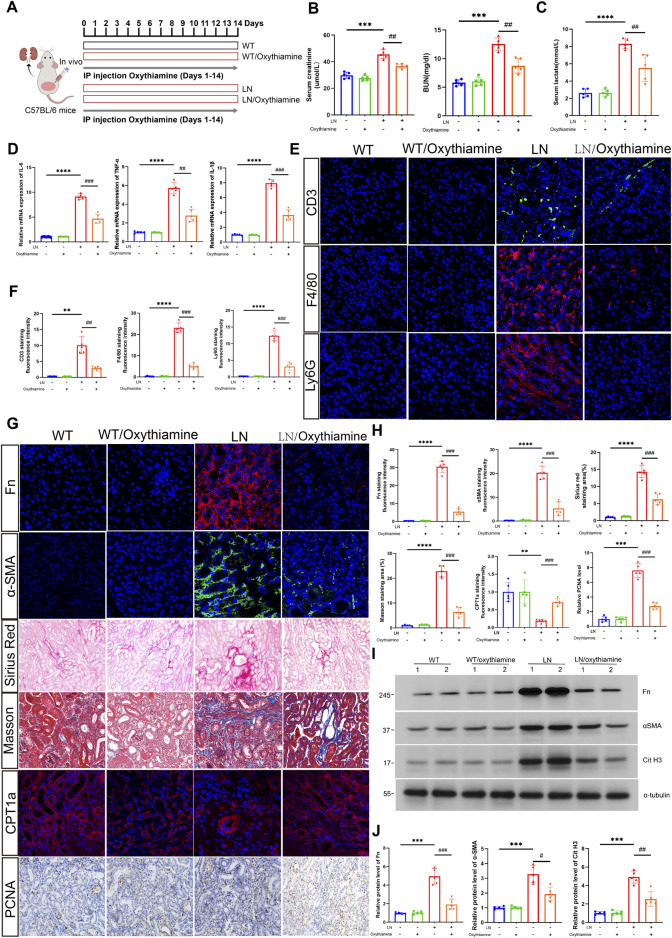
Oxythiamine treatment attenuates renal injury and NET-related inflammation in experimental lupus nephritis. **(A)** Schematic diagram of the *in vivo* experimental design. Wild-type (WT) and lupus nephritis (LN) mice were either left untreated or received intraperitoneal injections of the TKT inhibitor oxythiamine for 14 days **(B,C)** Serum creatinine and blood urea nitrogen (BUN) levels, and serum lactate concentrations, showing improved renal function and suppressed lactate accumulation in oxythiamine-treated LN mice. **(D)** Quantitative reverse transcription PCR (RT-qPCR) analysis revealing significantly decreased renal mRNA expression of pro-inflammatory cytokines (IL-6, TNF-α, and IL-1β) following oxythiamine treatment. **(E,F)** Representative immunofluorescence images and quantification demonstrating reduced renal infiltration of T cells (CD3), macrophages (F4/80), and neutrophils (Ly6G) in treated LN mice **(G,H)** Representative immunofluorescence, histological, and immunohistochemistry images with corresponding quantitative analyses. Oxythiamine treatment significantly ameliorates renal fibrosis, evidenced by decreased fluorescence intensity of fibronectin (Fn) and α-SMA, along with reduced Sirius Red and Masson’s trichrome staining areas. Furthermore, oxythiamine restores renal lipid metabolism, indicated by upregulated CPT1a expression, and suppresses aberrant cellular proliferation, indicated by downregulated PCNA levels. **(I,J)** Representative Western blot images and densitometric quantification showing that oxythiamine treatment was associated with reduced protein expression of TKT, ITGAM, the NET-associated marker Cit-H3, and fibrotic markers (Fn, α-SMA) in LN kidneys. Data are presented as mean ± SD. **P < 0.001, ****P < 0.0001 compared with the WT group; ^#^P < 0.05, ^##^P < 0.01, ^###^P < 0.001, ^####^P < 0.0001 compared with the untreated LN group (One-way ANOVA followed by Tukey’s *post hoc* test).

To explore the potential contribution of TKT-associated metabolism to lupus nephritis, we treated LN mice with the TKT inhibitor oxythiamine (Figure 12A). Oxythiamine treatment was associated with improved renal function, marked by reduced serum creatinine and BUN levels, which coincided with a substantial decrease in lactate accumulation (Figures 12B,C).

These changes were accompanied by reduced inflammatory responses. RT-qPCR revealed suppressed mRNA expression of IL-6, TNF-α, and IL-1β (Figure 12D), while immunofluorescence confirmed a drastic reduction in the intrarenal infiltration of CD3^+^ T cells, F4/80+ macrophages, and Ly6G + neutrophils (Figures 12E,F). To further evaluate NET-associated neutrophilic inflammatory activity, we performed Cit-H3/Ly6G co-immunofluorescence staining in renal tissues and measured plasma MPO-DNA complex levels. Cit-H3+Ly6G + double-positive signals were markedly increased in LN kidneys compared with WT controls, whereas oxythiamine treatment reduced these signals (Supplementary Figures S4A,B). In parallel, plasma MPO-DNA complex levels were elevated in LN mice and decreased after oxythiamine treatment (Supplementary Figure S4C). These findings provide additional evidence that oxythiamine treatment was associated with reduced NET-associated neutrophilic inflammatory activity in experimental LN.

Furthermore, oxythiamine treatment significantly ameliorated renal structural remodeling. We observed diminished expression of fibronectin and α-SMA, alongside a contraction of Sirius Red and Masson’s positive areas (Figures 12G,H). Notably, TKT inhibition also restored lipid metabolism (CPT1a upregulation) and suppressed aberrant cellular proliferation (PCNA downregulation). At the molecular level, Western blot analysis showed lower levels of ITGAM, fibrotic markers, and Cit-H3, a NET-associated marker, in oxythiamine-treated LN kidneys (Figures 12I,J). These data support a link between TKT-associated metabolic remodeling, inflammatory recruitment, and tissue injury in experimental LN.

## Discussion

It is increasingly recognized that lactic acid metabolism is not only involved in energy generation, but also in modulation of immune response. NETs released by activated neutrophils are involved in both tissue damage and inflammation in SLE. Growing evidence has suggested a link between lactate metabolism and NETs, where metabolism of lactic acid may augment NETs release through their effects on neutrophil activation and the downstream signal transduction pathways (Sun et al., 2024; Angeletti et al., 2021). Through exploring genes related to lactic acid metabolism and NETs, we identified ITGAM and TKT to be significantly altered in SLE and their potential as biomarker for predicting SLE development. Our results from immune cell profiling, functional analysis and cellular trajectory analysis all suggested the pathogenic relevance of ITGAM and TKT in SLE. Our additional analysis on human LN renal biopsy dataset (GSE32591) that showed upregulation of ITGAM and TKT in LN glomerular samples provided additional tissue-level evidence that these candidate genes are also dysregulated in human LN kidneys.

The product of ITGAM - integrin-αM (CD11b+) has been implicated in various immune responses, especially neutrophil adhesion and migration. CD11b+ couples with integrin-β2 to form a leukocyte-specific integrin, which exhibits a crucial role in neutrophils and monocytes adhesion to activated endothelium and phagocytosis of complement-coated particles. Previous studies also suggested that neutrophils from SLE patients with highly active disease showed increased CD11b/CD18 compared with patients with less active disease or healthy controls (Molad et al., 1994). One large GWAS study reported single nucleotide polymorphisms (SNPs) in ITGAM was associated with SLE and nephritis in Chinese and Thai populations (Yang et al., 2009). Furthermore, an imbalance between NET formation and clearance in SLE patients perpetuates autoimmunity and causes end-organ manifestations (Yu and Su, 2013). Our group recently also reported that dysregulated ITGAM was associated with NETs and inflammation in diabetic kidney disease (Xie et al., 2024). TKT and lactate are linked via the pentose phosphate pathway, and their dysregulation has been implicated in immune related disorders including lupus (Zhang et al., 2022; Tong et al., 2024). TKT, a master regulator of the pentose phosphate pathway, was induced in T cells for maintaining redox homeostasis and meeting the energetic requirements of activated immune cells. This pathway can be dysregulated (e.g., loss of aerobic glycolysis) and can contribute to an overall metabolic reprogramming in SLE, linking immune cell function to disease progression. Our results are consistent with previous studies demonstrating that metabolic reprogramming of immune cells modifies their functional programs and assume pathogenic roles in autoimmune disease (Mohammadnezhad et al., 2022; Blanco and Kaplan, 2023).

Our present findings indicated that the “DaCosta UV response via ERCC3 XPCS DN,” - a pathway which affects the cellular response to oxidative stress was differentially enriched, suggesting a connection between lactate metabolism, immune dysregulation in SLE. Our data also identified transcription factors and miRNAs relevant to ITGAM and TKT, elucidating their regulatory cross-talk in SLE pathogenesis. Previous investigators have also reported miRNA regulation in SLE and their possible involvement in regulating TKT and ITGAM (Navarro Quiroz et al., 2019).

Our scRNA-seq analysis suggested that ITGAM and TKT showed cell-type-associated expression patterns in SLE. After using donor-level summaries to reduce pseudoreplication, the most consistent donor-level signal was higher TKT expression in monocytes from SLE patients, whereas other ITGAM and TKT comparisons did not reach statistical significance. These findings should therefore be interpreted as exploratory and require validation in larger single-cell cohorts. Analysis of cell-cell communication revealed an orchestrating role of immune responses by T cells and its dynamic cross-talk with B cells and other immune cell subsets. Also, the expression of ITGAM and TKT changes with the cellular state of T cells. How such alterations in ITGAM and TKT expression may influence cellular maturation and function in SLE needs further investigations.

While bioinformatic analyses provided the initial landscape of metabolic dysregulation in SLE, our study extends these associations through rigorous *in vivo* functional validation. By employing an apoptotic cell-induced LN mouse model, we confirmed that the upregulation of the TKT-ITGAM axis is a consistent feature of lupus-associated renal injury, transcending beyond systemic circulation to localized tissue damage (Figure 11).

The oxythiamine intervention studies further extended our bioinformatic observations, showing that pharmacological inhibition of TKT was accompanied by attenuation of renal injury, inflammatory cytokine expression, and NET-related activity in experimental LN. Because TKT functions in the non-oxidative branch of the pentose phosphate pathway rather than directly catalyzing glycolytic lactate production, the reduced serum lactate level after oxythiamine treatment should be interpreted as an indirect metabolic association. This may reflect changes in pentose phosphate pathway–glycolytic carbon flux, redox balance, immune-cell activation, and the overall inflammatory burden within nephritic kidneys. Therefore, the observed reductions in serum lactate, inflammatory cytokine expression, and Cit-H3 levels are consistent with a relationship between TKT-associated metabolic remodeling and reduced inflammatory activity, rather than a direct TKT-catalyzed pathway of lactate production (Figure 12). Furthermore, the altered CPT1a expression and reduced fibrotic marker levels suggest that oxythiamine treatment may be linked to broader metabolic and structural improvement in nephritic kidneys.

Although our scRNA-seq data were derived from PBMCs, the renal biopsy dataset analysis and mouse kidney validation, including renal IgG/C3 deposition and TKT staining in CD14^+^ monocyte-lineage cells, provided tissue-level support for the relevance of these immune signatures to renal involvement (Figures 11J,N). This cross-validation suggests that immune signatures identified in peripheral blood are, at least in part, reflected within the nephritic kidney microenvironment.

Several limitations should be acknowledged when interpreting our findings. First, the primary discovery and validation datasets came from peripheral blood samples of SLE cohorts which mainly reflected lupus-associated systemic immune signatures rather than mechanisms specific to LN-related kidney injury. Furthermore, detailed clinicopathological metadata, including renal function, proteinuria, histological classification (including activity/chronicity indices) were not available in the GSE32591 data used for kidney-level validation. Second, the MR analysis should be interpreted as supportive genetic evidence rather than definitive proof of causality. The instrumental variables were selected using a relatively relaxed threshold, and the number of SNPs available for some genes was limited. Larger GWAS datasets, stronger tissue- or cell-type-specific eQTL instruments, and additional colocalization analyses in relevant tissues or immune-cell populations are needed to further clarify the genetic relationship between TKT, ITGAM, and SLE. Third, the regulatory networks involving ITGAM, TKT, transcription factors, and microRNAs were generated from public prediction databases and should be interpreted as bioinformatic hypotheses rather than experimentally confirmed molecular mechanisms. Although oxythiamine treatment was accompanied by lower lactate levels, reduced ITGAM expression, less inflammatory cell infiltration, and decreased Cit-H3 expression in nephritic kidneys, these observations did not establish causal relationships between TKT activity, lactate metabolism or lactylation, ITGAM regulation, NET-associated inflammatory activity, and renal protection. While Cit-H3/Ly6G co-immunofluorescence staining and plasma MPO-DNA complex measurement provided additional support for reduced NET-associated neutrophilic inflammatory activity after oxythiamine treatment, these findings did not form a causal link between TKT activity, lactate metabolism, NET-associated inflammation, and renal protection. Cit-H3 is not a NET-specific marker, and changes in Cit-H3/Ly6G signals may partly reflect altered neutrophil infiltration, histone citrullination, or the overall degree of renal inflammation. Since the intervention was based on pharmacological inhibition, possible effects of oxythiamine on other thiamine-dependent metabolic pathways cannot be completely excluded. Further work using cell-specific TKT knockdown or overexpression, direct assessment of TKT enzymatic activity, intracellular lactate and protein or histone lactylation, as well as lactate-rescue experiments and blockade of ITGAM or NET formation, will be needed to elucidate how these events are mechanistically connected in lupus nephritis.

## Conclusion

In summary, our study indicates that TKT-associated immunometabolic remodeling is linked to the inflammatory kidney microenvironment in experimental LN. In this model, oxythiamine treatment attenuated renal injury and inflammatory remodeling, accompanied by reduced lactate accumulation, ITGAM expression, immune-cell infiltration, and NET-related activity. These findings support the potential relevance of TKT as an immunometabolic target in LN, while further studies are needed to clarify the cell-specific mechanisms connecting TKT activity, lactate metabolism or lactylation, ITGAM regulation, and NET-associated inflammatory activity.

## Data Availability

The datasets analyzed in this study are publicly available in the Gene Expression Omnibus (GEO) database (https://www.ncbi.nlm.nih.gov/geo/). The accession numbers are GSE50772, GSE72326, GSE135779, and GSE32591.
