# Supplementary material for: Targeting TKT-associated immunometabolic remodeling attenuates experimental lupus nephritis and NET-related inflammation
Source: Front Cell Dev Biol. 2026 Jul 1;14:1835407. doi: 10.3389/fcell.2026.1835407 (PMC13370341; doi:10.3389/fcell.2026.1835407)
Supplement: Supplementary file 1 [file DataSheet1.zip › Supplementary_Materials_final version/Supplementary Figures.docx]

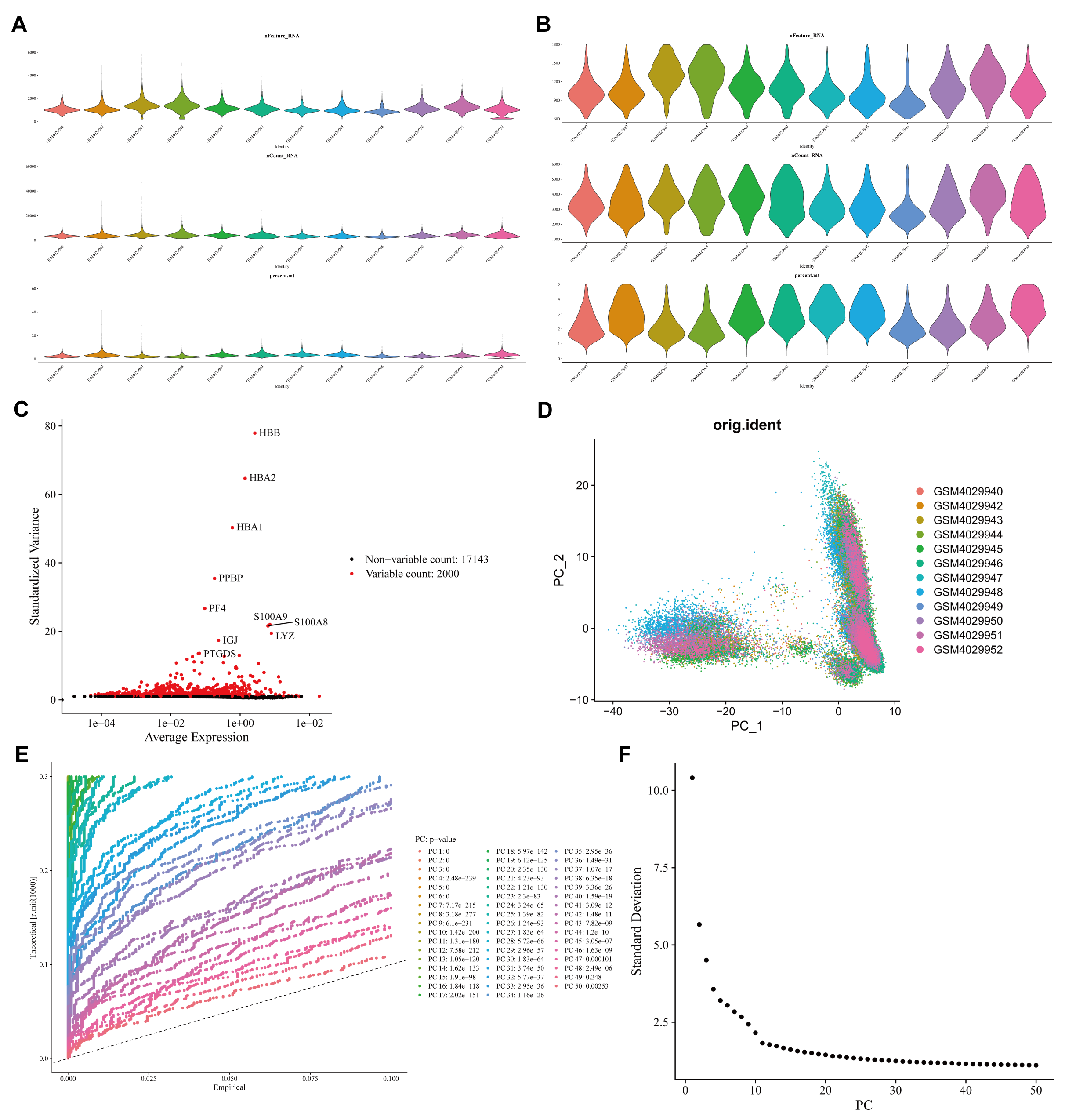


**Supplementary Figure S1.** Quality control and dimension reduction of scRNA-seq data. (A-B) Visualization of nFeature, nCount, and mitochondrial content before and after quality control. (C) Selection of 2,000 highly variable genes. (D-F) PCA analysis and selection of the top 30 principal components for downstream analysis.

**
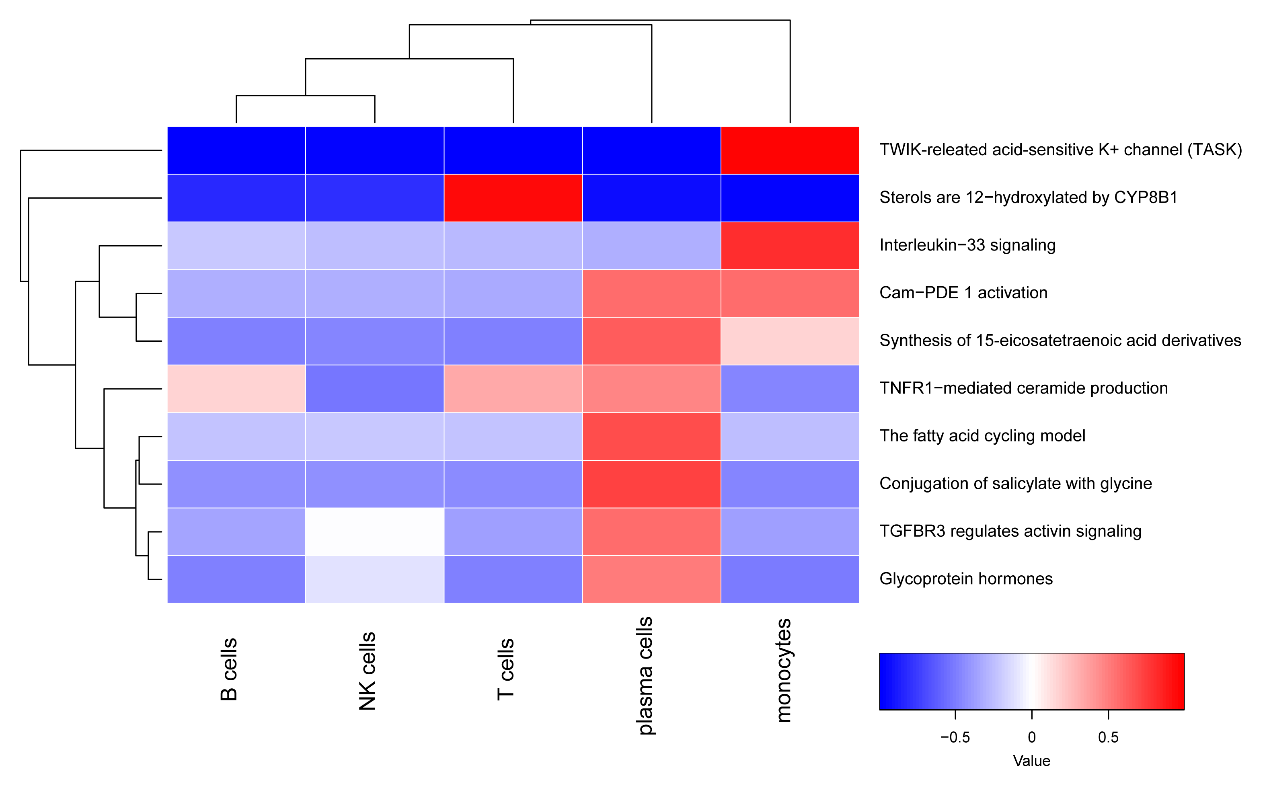
**

**Supplementary Figure S2.** Global functional profiling of the identified immune cell subpopulations. Heatmap illustrating distinct pathway enrichment signatures across the five major cell lineages. Representative highly enriched terms include TWIK-related acid-sensitive K+ channel activity, CYP8B1-mediated sterol hydroxylation, salicylate-glycine conjugation, IL-33 signaling, and glycoprotein hormone responses, underscoring the baseline metabolic and inflammatory heterogeneity among these cell types.


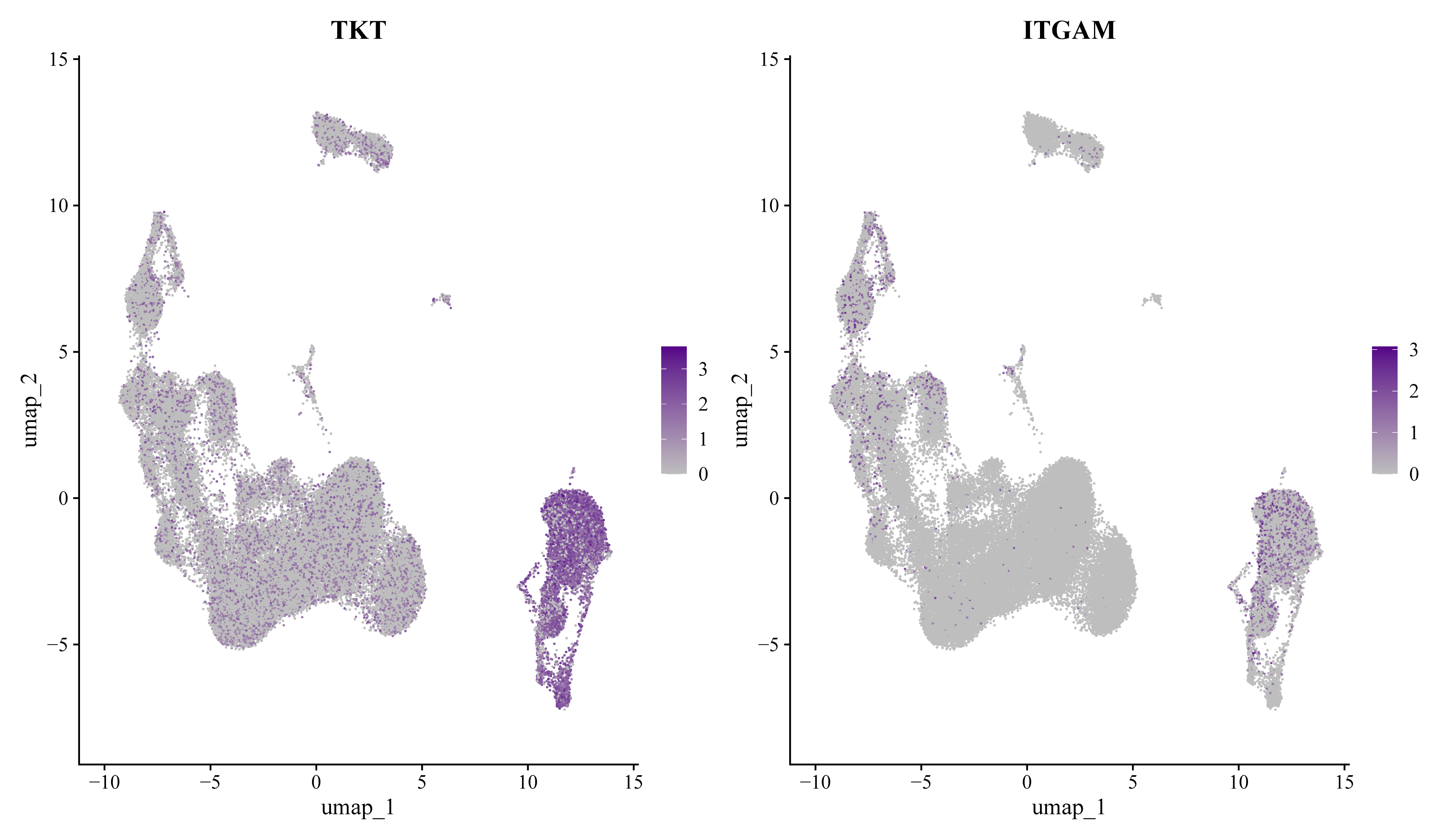


**Supplementary Figure S3.** Feature plots illustrating the expression distribution of ITGAM and TKT. UMAP plots visualizing the expression intensity and spatial distribution of ITGAM and TKT across all annotated cell clusters. The purple gradient indicates the expression level of the respective genes.


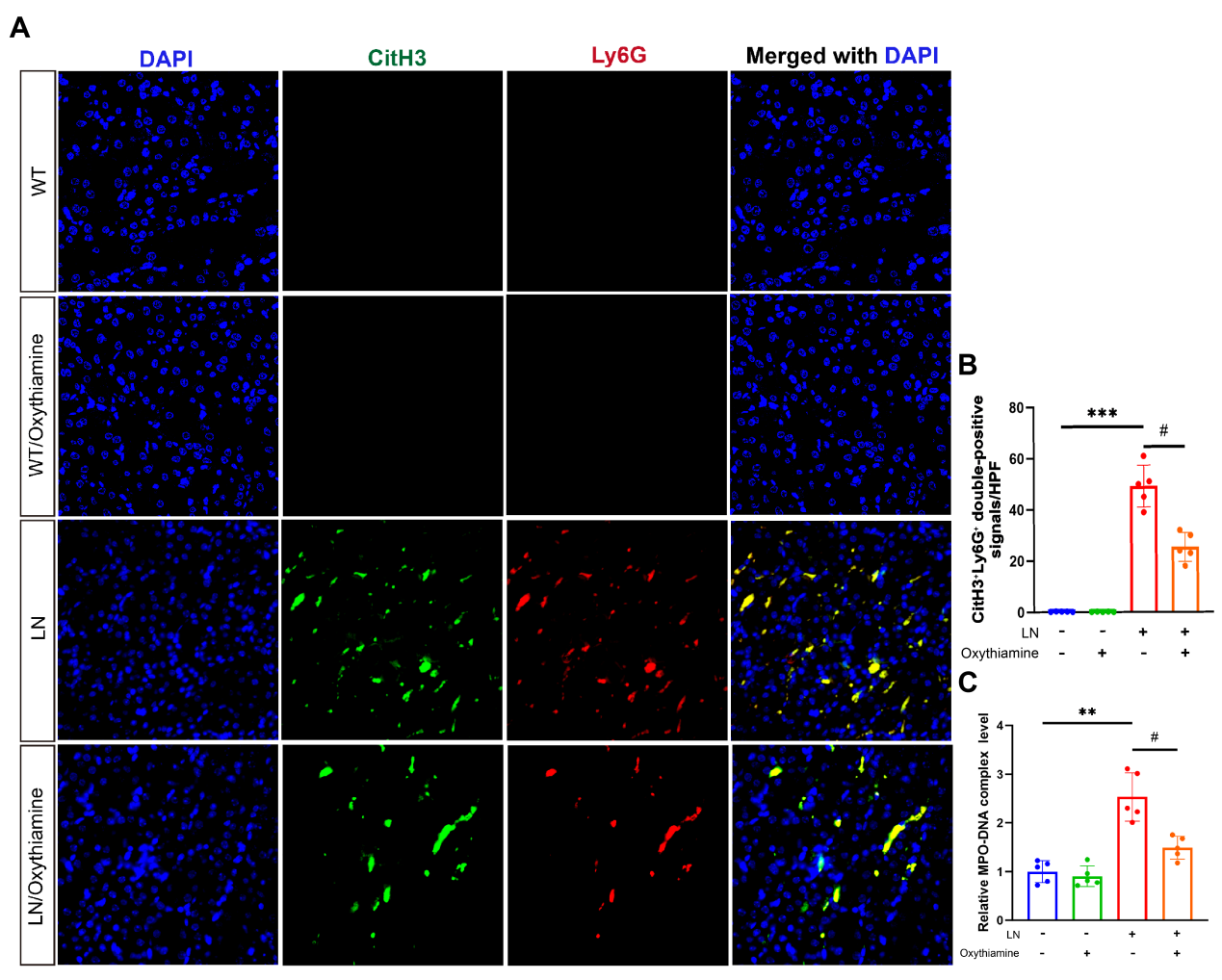


**Supplementary Figure S4.** Oxythiamine treatment reduces NET-associated neutrophilic signals in experimental lupus nephritis.

**(A)** Representative immunofluorescence images showing DAPI (blue), Cit-H3 (green), Ly6G (red), and merged staining in kidney sections from WT, WT + oxythiamine, LN, and LN + oxythiamine mice. Cit-H3/Ly6G double-positive signals were increased in LN kidneys and were reduced after oxythiamine treatment.

**(B)** Quantification of Cit-H3+Ly6G+ double-positive signals per high-power field (HPF), showing increased renal Cit-H3/Ly6G co-localized signals in LN mice and their reduction after oxythiamine treatment.

**(C)** Plasma MPO-DNA complex levels were measured as an additional NET-associated marker. MPO-DNA complex levels were increased in LN mice and decreased following oxythiamine treatment. Values were normalized to the mean value of the WT group.

Data are presented as mean ± SD; n = 5 mice per group. **P < 0.01 and ***P < 0.001 versus WT; #P < 0.05 versus untreated LN. Statistical analysis was performed using one-way ANOVA followed by Tukey’s post hoc test.
