# Supplementary material for: Targeting TKT-associated immunometabolic remodeling attenuates experimental lupus nephritis and NET-related inflammation
Source: Front Cell Dev Biol. 2026 Jul 1;14:1835407. doi: 10.3389/fcell.2026.1835407 (PMC13370341; doi:10.3389/fcell.2026.1835407)
Supplement: Supplementary file 1 [file DataSheet1.zip › Supplementary_Materials_final version/Supplementary Tables.docx]

**Supplementary Table S1.** The lists of Lactic Acid Metabolism-Related Genes (LAM-RGs) and Neutrophil Extracellular Traps-Related Genes (NETs-RGs). (This table is provided as a separate Excel file: Supplementary Table S1.xlsx)

**Supplementary Table S2.** The list of gene symbols and their corresponding ENSEMBL IDs.

| SYMBOL | ENSEMBL |
| --- | --- |
| MPO | eqtl-a-ENSG00000005381 |
| MMP9 | eqtl-a-ENSG00000100985 |
| MAPK3 | eqtl-a-ENSG00000102882 |
| PIK3CG | eqtl-a-ENSG00000105851 |
| PPARGC1A | eqtl-a-ENSG00000109819 |
| FN1 | eqtl-a-ENSG00000115414 |
| IL1B | eqtl-a-ENSG00000125538 |
| TLR2 | eqtl-a-ENSG00000137462 |
| ALPL | eqtl-a-ENSG00000162551 |
| NLRP3 | eqtl-a-ENSG00000162711 |
| CXCR1 | eqtl-a-ENSG00000163464 |
| TKT | eqtl-a-ENSG00000163931 |
| ITGAM | eqtl-a-ENSG00000169896 |
| CEACAM3 | eqtl-a-ENSG00000170956 |
| THBD | eqtl-a-ENSG00000178726 |
| CXCR2 | eqtl-a-ENSG00000180871 |
| ANXA2 | eqtl-a-ENSG00000182718 |
| MME | eqtl-a-ENSG00000196549 |
| SRC | eqtl-a-ENSG00000197122 |
| C5AR1 | eqtl-a-ENSG00000197405 |
| ELANE | eqtl-a-ENSG00000197561 |
| TNF | eqtl-a-ENSG00000232810 |
| MGAM | eqtl-a-ENSG00000257335 |
| CCL2 | eqtl-a-ENSG00000108691 |
| CCL4 | eqtl-a-ENSG00000275302 |
| CFP | eqtl-a-ENSG00000126759 |
| CFTR | eqtl-a-ENSG00000001626 |
| TIMP1 | eqtl-a-ENSG00000102265 |

*Note: IDs were retrieved from the IEU OpenGWAS project database.*

**Supplementary Table S3:** Primers for real time-quantitative PCR.

| Gene | Direction | Sequence(5’ to 3’) |
| --- | --- | --- |
| ITGAM | Forward | 5'-AGTGTGAAGCTCTTCTCCACG-3' |
|  | Reverse | 5'-AAAATGTAGACAGCGCCCTGA-3' |
| TKT | Forward | 5'-GCTAACATCCGAATGCCTACGC-3' |
|  | Reverse | 5'-TTGGTGTCTCCATCCAGGGCAA-3' |
| IL-6 | Forward | 5'-AGGAGACTTCACAGAGGATACCA-3' |
|  | Reverse | 5'-TTCCACGATTTCCCAGAGAACAT-3' |
| TNF-α | Forward | 5'-CCCAGGTTCTCTTCAAGGGAC-3' |
|  | Reverse | 5'-CTGGAAGACTCCTCCCAGGTA-3' |
| IL‑1β | Forward | 5'-AACCTTTGACCTGGGCTGTC-3' |
|  | Reverse | 5'-AAGGTCCACGGGAAAGACAC-3' |
| β-actin | Forward | 5'-GAGCGCAAGTACTCTGTGTG-3' |
|  | Reverse | 5'-AACGCAGCTCAGTAACAGTC-3' |

**Supplementary Table S4.** The detailed results of GO and KEGG enrichment analyses. (This table is provided as a separate Excel file: Supplementary Table S4.xlsx, containing two sheets: "GO Analysis" and "KEGG Analysis")

**Supplementary Table S5.** The Degree centrality values of the hub genes in the Protein-Protein Interaction (PPI) network.

| gene name | Degree |
| --- | --- |
| TNF | 24 |
| IL1B | 23 |
| ITGAM | 20 |
| MMP9 | 19 |
| MPO | 18 |
| CCL2 | 18 |
| TLR2 | 18 |
| SRC | 17 |
| MAPK3 | 16 |
| CXCR2 | 16 |
| ELANE | 16 |
| FN1 | 15 |
| C5AR1 | 15 |
| NLRP3 | 15 |
| TIMP1 | 14 |
| CXCR1 | 14 |
| CCL4 | 14 |
| THBD | 11 |
| CFP | 10 |
| MME | 6 |
| PPARGC1A | 6 |
| PIK3CG | 6 |
| ANXA2 | 5 |
| CFTR | 4 |
| MGAM | 2 |
| ALPL | 1 |
| CEACAM3 | 1 |

Note: The degree values were calculated using the CytoHubba plugin in Cytoscape software to identify hub genes.

**Supplementary Table S6.** The number of Single Nucleotide Polymorphisms (SNPs) used as instrumental variables for each hub gene in Mendelian Randomization analysis.

| NO. | id | nsnp | SYMBOL |
| --- | --- | --- | --- |
| 1 | eqtl-a-ENSG00000005381 | 17 | MPO |
| 2 | eqtl-a-ENSG00000100985 | 20 | MMP9 |
| 3 | eqtl-a-ENSG00000102882 | 13 | MAPK3 |
| 4 | eqtl-a-ENSG00000105851 | 5 | PIK3CG |
| 5 | eqtl-a-ENSG00000109819 | 12 | PPARGC1A |
| 6 | eqtl-a-ENSG00000115414 | 5 | FN1 |
| 7 | eqtl-a-ENSG00000125538 | 12 | IL1B |
| 8 | eqtl-a-ENSG00000137462 | 4 | TLR2 |
| 9 | eqtl-a-ENSG00000162551 | 13 | ALPL |
| 10 | eqtl-a-ENSG00000162711 | 19 | NLRP3 |
| 11 | eqtl-a-ENSG00000163464 | 14 | CXCR1 |
| 12 | eqtl-a-ENSG00000163931 | 20 | TKT |
| 13 | eqtl-a-ENSG00000169896 | 9 | ITGAM |
| 14 | eqtl-a-ENSG00000170956 | 8 | CEACAM3 |
| 15 | eqtl-a-ENSG00000178726 | 20 | THBD |
| 16 | eqtl-a-ENSG00000180871 | 8 | CXCR2 |
| 17 | eqtl-a-ENSG00000182718 | 12 | ANXA2 |
| 18 | eqtl-a-ENSG00000196549 | 17 | MME |
| 19 | eqtl-a-ENSG00000197122 | 8 | SRC |
| 20 | eqtl-a-ENSG00000197405 | 6 | C5AR1 |
| 21 | eqtl-a-ENSG00000197561 | 10 | ELANE |
| 22 | eqtl-a-ENSG00000232810 | 16 | TNF |
| 23 | eqtl-a-ENSG00000257335 | 4 | MGAM |

Note: "nsnp" refers to the number of independent SNPs selected as instrumental variables for each gene. IDs represent the dataset identifiers from the IEU OpenGWAS database.

**Supplementary Table S7.** The results of the Mendelian Randomization (MR) analysis using the Inverse Variance Weighted (IVW) method. (This table is provided as a separate Excel file: Supplementary Table S7.xlsx).

**Supplementary Table S8.** The results of the heterogeneity test for Mendelian Randomization analysis.

| No. | id | heterogeneity p value | SYMBOL |
| --- | --- | --- | --- |
| 1 | eqtl-a-ENSG00000178726 | 0.132200893 | THBD |
| 2 | eqtl-a-ENSG00000163931 | 0.584177676 | TKT |
| 3 | eqtl-a-ENSG00000169896 | 0.134431375 | ITGAM |

Note: A Heterogeneity P-value > 0.05 indicates no significant heterogeneity among the instrumental variables.

**Supplementary Table S9.** The results of the horizontal pleiotropy test (MR-Egger intercept) for Mendelian Randomization analysis.

| No. | id | pleiotropy p value | SYMBOL |
| --- | --- | --- | --- |
| 1 | eqtl-a-ENSG00000178726 | 0.711235970 | THBD |
| 2 | eqtl-a-ENSG00000163931 | 0.487902113 | TKT |
| 3 | eqtl-a-ENSG00000169896 | 0.580021311 | ITGAM |

Note: A Pleiotropy P-value > 0.05 indicates no significant horizontal pleiotropy, suggesting the results are not biased by confounding factors.

**Supplementary Table S10.** The results of the Steiger directionality test for Mendelian Randomization analysis.

| No. | id | steiger dir | steiger p value | SYMBOL |
| --- | --- | --- | --- | --- |
| 1 | eqtl-a-ENSG00000163931 | TRUE | 0 | TKT |
| 2 | eqtl-a-ENSG00000169896 | TRUE | 8.63E-46 | ITGAM |
| 3 | eqtl-a-ENSG00000178726 | TRUE | 4.30E-223 | THBD |

Note: A "True" directionality indicates that the instrumental variables explain significantly more variance in the exposure than in the outcome, confirming the direction of causality from exposure to outcome.

**Supplementary Table S11**. FDR-corrected results of the heterogeneity test for Mendelian randomization analysis.
(This table is provided as a separate CSV file: Supplementary Table S11.csv)

**Supplementary Table S12**. FDR-corrected results of the horizontal pleiotropy test for Mendelian randomization analysis.
(This table is provided as a separate CSV file: Supplementary Table S12.csv)

**Supplementary Table S13**. FDR-corrected results of the Steiger directionality test for Mendelian randomization analysis.
(This table is provided as a separate CSV file: Supplementary Table S13.csv)

**Supplementary Table S14.** The results of Gene Set Variation Analysis (GSVA). (This table is provided as a separate Excel file: Supplementary Table S14.xlsx)

**Supplementary Table S15.** The detailed results of immune infiltration correlation analysis. (This table is provided as a separate Excel file: Supplementary Table S15.xlsx, containing two sheets: "Gene-Immune Correlation" and "Immune-Immune Correlation")

**Supplementary Table S16.** The regulatory network of transcription factors and miRNAs targeting the hub genes. (This table is provided as a separate Excel file: Supplementary Table S16.xlsx, containing two sheets: "TF-mRNA Interaction" and "mRNA-miRNA Interaction")
