# Supplementary figures and images for: Targeting TKT-associated immunometabolic remodeling attenuates experimental lupus nephritis and NET-related inflammation
Source: Front Cell Dev Biol. 2026 Jul 1;14:1835407. doi: 10.3389/fcell.2026.1835407 (PMC13370341; doi:10.3389/fcell.2026.1835407)

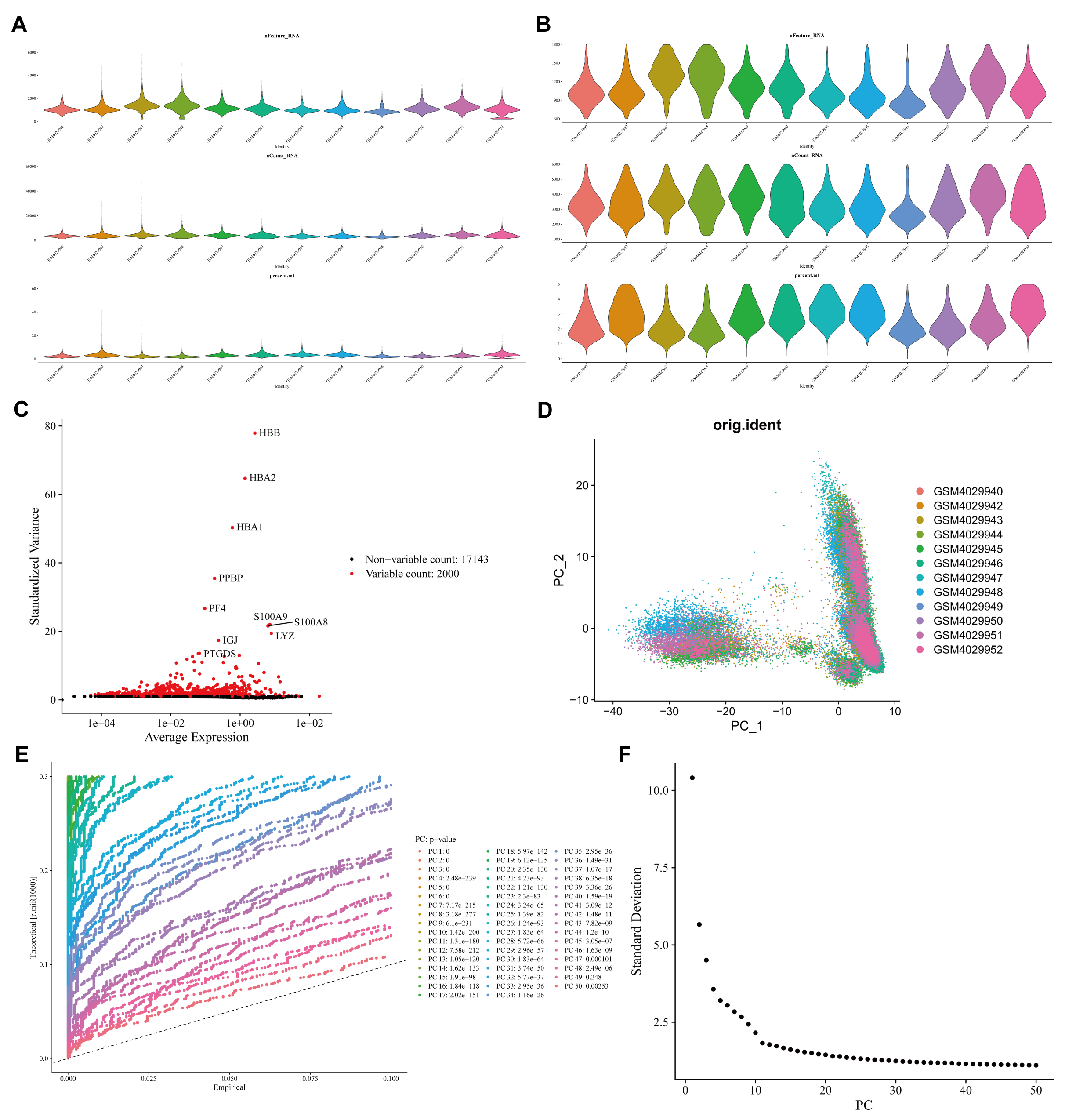

Supplement: Supplementary file 1 [file DataSheet1.zip › Supplementary_Materials_final version/Supplementary Figure S1.tif]

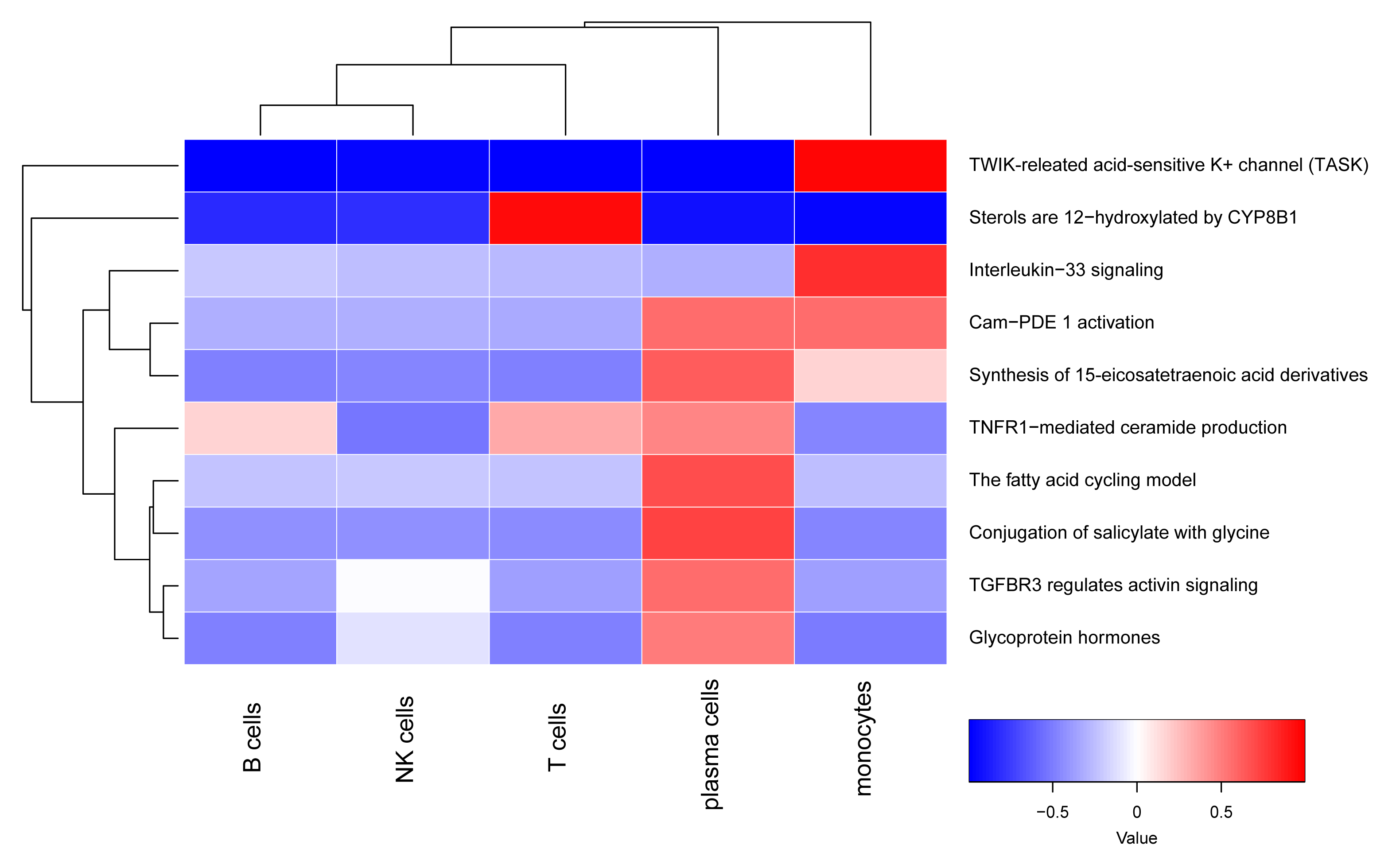

Supplement: Supplementary file 1 [file DataSheet1.zip › Supplementary_Materials_final version/Supplementary Figure S2.tif]

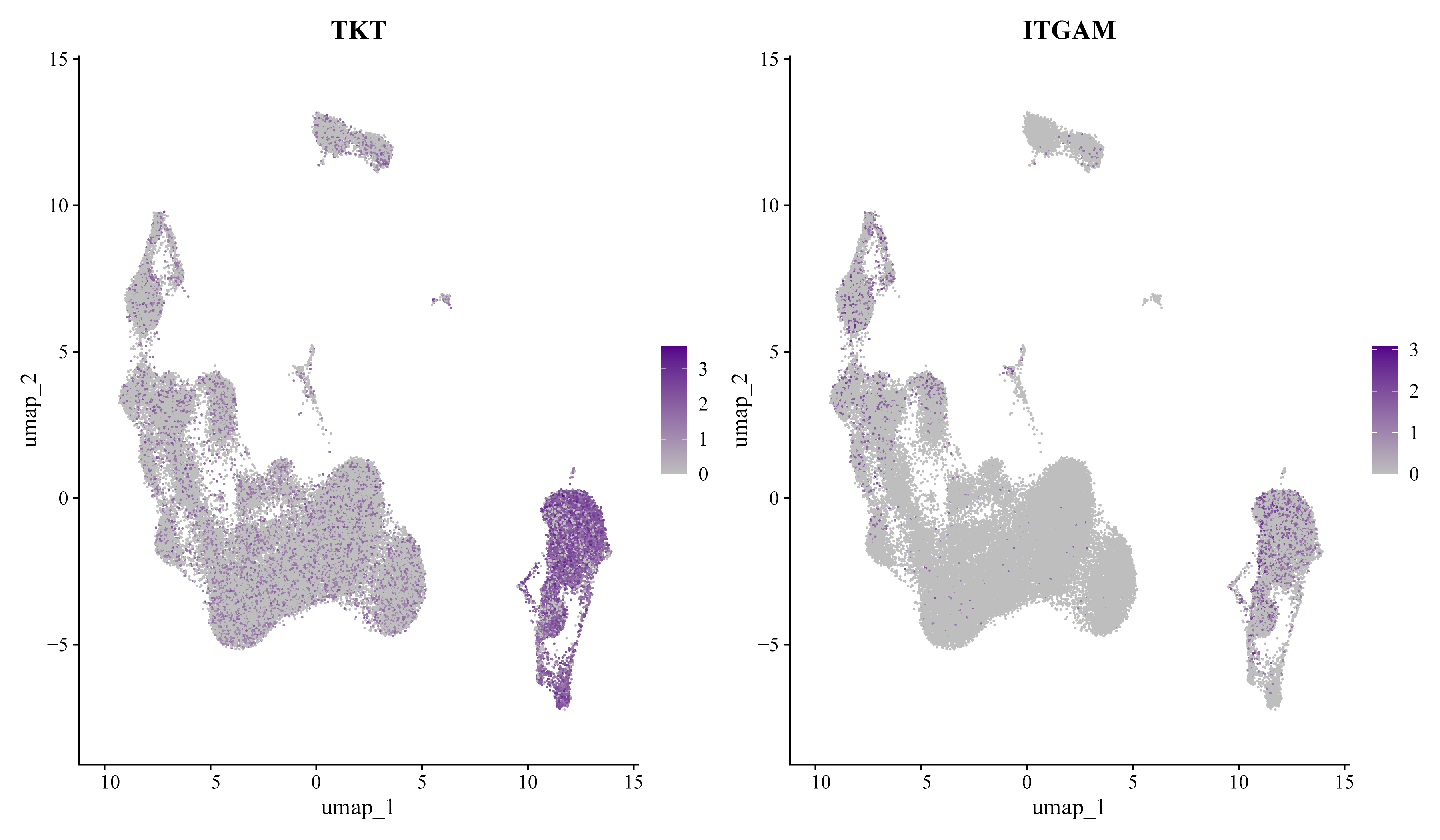

Supplement: Supplementary file 1 [file DataSheet1.zip › Supplementary_Materials_final version/Supplementary Figure S3.tif]

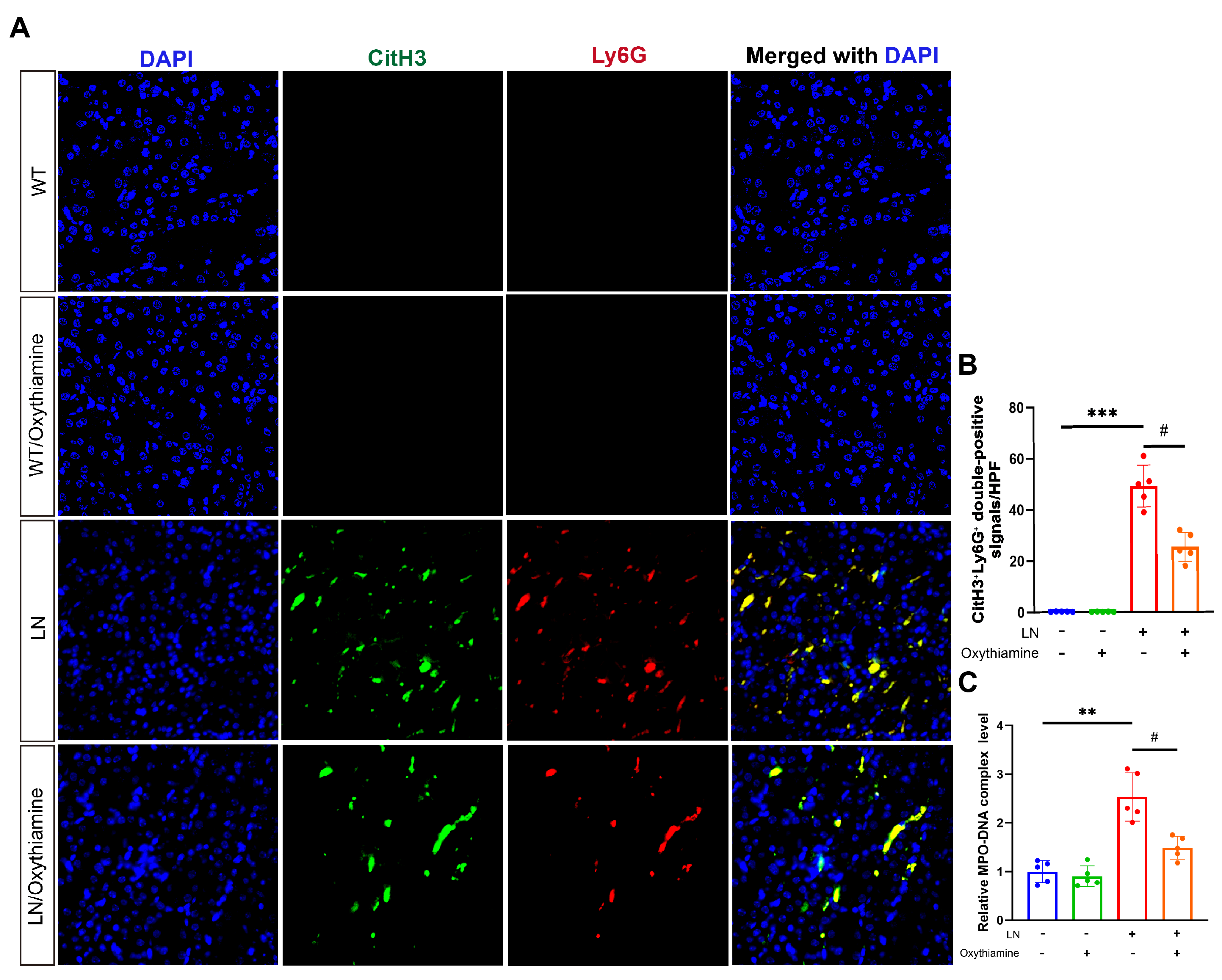

Supplement: Supplementary file 1 [file DataSheet1.zip › Supplementary_Materials_final version/Supplementary Figure S4.tif]
